# Activating transcription factor 4 (ATF4) promotes skeletal muscle atrophy by forming a heterodimer with the transcriptional regulator C/EBPβ

**DOI:** 10.1074/jbc.RA119.012095

**Published:** 2020-01-17

**Authors:** Scott M. Ebert, Steven A. Bullard, Nathan Basisty, George R. Marcotte, Zachary P. Skopec, Jason M. Dierdorff, Asma Al-Zougbi, Kristin C. Tomcheck, Austin D. DeLau, Jacob A. Rathmacher, Sue C. Bodine, Birgit Schilling, Christopher M. Adams

**Affiliations:** ‡ Departments of Internal Medicine and Molecular Physiology and Biophysics, Fraternal Order of Eagles Diabetes Research Center, University of Iowa, Iowa City, Iowa 52242; § Iowa City Veterans Affairs Medical Center, Iowa City, Iowa 52246; ¶ Emmyon, Inc., Coralville, Iowa 52241; ‖ Buck Institute for Research on Aging, Novato, California 94945

**Keywords:** muscle atrophy, skeletal muscle, muscle, protein–protein interaction, protein–DNA interaction, gene regulation, activating transcription factor 4 (ATF4), basic leucine zipper transcription factor (bZIP), CCAAT enhancer-binding protein β (C/EBPβ), growth arrest and DNA damage-inducible α (Gadd45α)

## Abstract

Skeletal muscle atrophy is a highly-prevalent and debilitating condition that remains poorly understood at the molecular level. Previous work found that aging, fasting, and immobilization promote skeletal muscle atrophy via expression of activating transcription factor 4 (ATF4) in skeletal muscle fibers. However, the direct biochemical mechanism by which ATF4 promotes muscle atrophy is unknown. ATF4 is a member of the basic leucine zipper transcription factor (bZIP) superfamily. Because bZIP transcription factors are obligate dimers, and because ATF4 is unable to form highly-stable homodimers, we hypothesized that ATF4 may promote muscle atrophy by forming a heterodimer with another bZIP family member. To test this hypothesis, we biochemically isolated skeletal muscle proteins that associate with the dimerization- and DNA-binding domain of ATF4 (the bZIP domain) in mouse skeletal muscle fibers *in vivo*. Interestingly, we found that ATF4 forms at least five distinct heterodimeric bZIP transcription factors in skeletal muscle fibers. Furthermore, one of these heterodimers, composed of ATF4 and CCAAT enhancer-binding protein β (C/EBPβ), mediates muscle atrophy. Within skeletal muscle fibers, the ATF4–C/EBPβ heterodimer interacts with a previously unrecognized and evolutionarily conserved ATF–C/EBP composite site in exon 4 of the *Gadd45a* gene. This three-way interaction between ATF4, C/EBPβ, and the ATF–C/EBP composite site activates the *Gadd45a* gene, which encodes a critical mediator of muscle atrophy. Together, these results identify a biochemical mechanism by which ATF4 induces skeletal muscle atrophy, providing molecular-level insights into the etiology of skeletal muscle atrophy.

## Introduction

Skeletal muscle atrophy impairs the health and quality of life of tens of millions of people. Its causes include aging, muscle disuse, malnutrition, and essentially any serious illness or injury. All of these conditions cause skeletal muscle fibers to become smaller, or atrophic, leading to a loss of skeletal muscle mass and strength (1). The molecular mechanisms of skeletal muscle atrophy are not yet well-understood. However, a few proteins have recently emerged as important mediators of muscle atrophy, and one of those proteins is activating transcription factor 4 (ATF4)^2^ (1).

ATF4 is a stress-inducible member of the basic leucine zipper transcription factor (bZIP) superfamily. Conditional knockout mice lacking ATF4 in skeletal muscle fibers develop normally, exhibit normal muscle mass and function as young and middle-aged adults, and demonstrate resistance to immobilization-induced muscle atrophy, fasting-induced muscle atrophy, and age-related muscle atrophy (2–5). Conversely, forced expression of ATF4 in skeletal muscle fibers is sufficient to induce muscle atrophy in the absence of aging or any acute stress condition (3–5).

ATF4 promotes muscle atrophy by increasing the levels of specific mRNAs in skeletal muscle fibers, most notably *Gadd45a* (*growth arrest and DNA damage-inducible 45* α) (2, 5). Skeletal muscle *Gadd45a* expression is low in young, healthy skeletal muscle but is strongly induced by aging and acute stress conditions in humans, mice, and other mammalian species (2, 5–12). ATF4 is necessary and sufficient for *Gadd45a* expression in skeletal muscle fibers, which is sufficient to induce muscle atrophy and necessary for ATF4-mediated muscle atrophy (2, 5, 13, 14).

The mechanism by which ATF4 increases mRNAs such as *Gadd45a* within muscle fibers is unknown. bZIP proteins such as ATF4 must dimerize to bind and activate genes (15–18). However, ATF4 is unable to form stable homodimers (19), and a heterodimerization partner of ATF4 in skeletal muscle has never been found. This represents an important gap in our understanding of how skeletal muscle atrophy occurs at the molecular level.

In non-muscle systems, ATF4 has a strong propensity to form heterodimers with many other bZIP family members. For example, *in vitro* analyses of interactions between human bZIP family members found that, compared with its affinity for another ATF4 bZIP domain, an individual ATF4 bZIP domain has a higher affinity for the bZIP domains of at least 30 other bZIP family members (20, 21). Consistent with this finding, ATF4 heterodimers are known to play important roles in nonmuscle cells (22–28), and several ATF4 target genes contain non-palindromic ATF4 regulatory elements, indicating regulation by an ATF4 heterodimer (29–31).

These considerations led us to hypothesize that ATF4 may promote skeletal muscle atrophy by heterodimerizing with another bZIP family member. To test this hypothesis, we conducted an unbiased search for ATF4 heterodimerization partners in mouse skeletal muscle fibers.

## Results

### Identification of proteins that interact with the ATF4 bZIP domain in mouse skeletal muscle fibers in vivo

**Figure 1.**
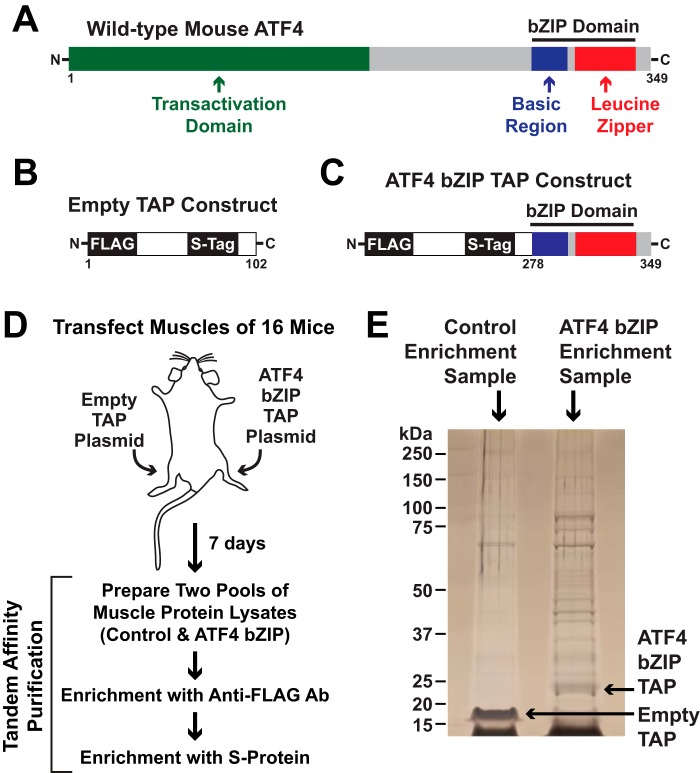
**Isolation of proteins that interact with the ATF4 bZIP domain in mouse skeletal muscle fibers.** *A–C*, schematic illustrations of ATF4 (*A*), the empty TAP construct (*B*), and the ATF4 bZIP TAP construct (*C*). *D,* tibialis anterior (TA) muscle fibers of 16 mice were transfected with 20 μg of empty TAP plasmid (one TA per mouse) or 20 μg of ATF4 bZIP TAP plasmid (the contralateral TA in each mouse). Seven days post-transfection, bilateral TA muscles were harvested and used to prepare pooled protein extracts from each of the two groups of skeletal muscles (control and ATF4 bZIP). The pooled protein extracts were then subjected to sequential purification steps with anti-FLAG magnetic beads and S-protein affinity gel. *E,* SDS-PAGE and silver staining of final pulldown samples.

We recently developed and validated an *in vivo* tandem affinity purification (TAP) method for proteomic identification of protein–protein interactions in mouse skeletal muscle fibers (14). Here, we used that same general approach to search for ATF4 heterodimerization partners in muscle fibers. The schematic in Fig. 1*A* shows full-length ATF4, which contains a basic leucine zipper (bZIP) domain close to the C terminus. In our initial attempts to identify ATF4 heterodimerization partners in skeletal muscle, we placed two affinity tags (FLAG and S-tag) at the N terminus of full-length ATF4 and then used that construct as bait in TAP experiments in mouse skeletal muscle fibers. However, in three independent experiments using full-length ATF4 as bait, we were unable to detect any evidence of ATF4 in the affinity enrichment samples. This suggested that full-length ATF4 may be too unstable in skeletal muscle fibers to be suitable for TAP, which would be consistent with previous findings that full-length ATF4 is highly unstable due to rapid degradation (31–36).

In ATF4, the bZIP domain comprises about 18% of the protein (Fig. 1*A*) and is necessary and sufficient for dimerization (19, 21, 37). The rapid degradation of ATF4 is mediated by portions of ATF4 that lie outside of the ATF4 bZIP domain (32–36). We therefore hypothesized that we might obtain a more stable and suitable TAP construct by eliminating the regions outside the bZIP domain and using only the ATF4 bZIP domain as bait. Fig. 1*B* shows our empty TAP construct, which contains two affinity tags, FLAG and S-tag, but no bait. To generate the ATF4 bZIP TAP construct, we fused the empty TAP construct to the N terminus of the ATF4 bZIP domain (Fig. 1*C*).

We then followed the strategy illustrated in Fig. 1*D*. Briefly, we used *in vivo* electroporation to transfect tibialis anterior (TA) muscle fibers of 16 mice with plasmid DNA encoding the ATF4 bZIP TAP construct. In each mouse, the contralateral TA was transfected with plasmid DNA encoding the empty TAP construct. Following transfection, the mice returned to their normal activities for 1 week. Importantly, *in vivo* electroporation transfects differentiated muscle fibers, but not satellite cells or connective tissue cells (38). Expression of transfected plasmids in mouse skeletal muscle fibers occurs within 3 days and continues at a high level for at least 10 weeks (Fig. S1).

One week after transfection, we euthanized the mice and harvested bilateral TA muscles from each mouse. We then prepared and pooled protein extracts from the two groups of TA muscles (control and ATF4 bZIP) and subjected the pooled extracts to sequential purification steps with anti-FLAG magnetic beads and S-protein agarose. We performed two identical but independent experiments in this manner.

**Figure 2.**
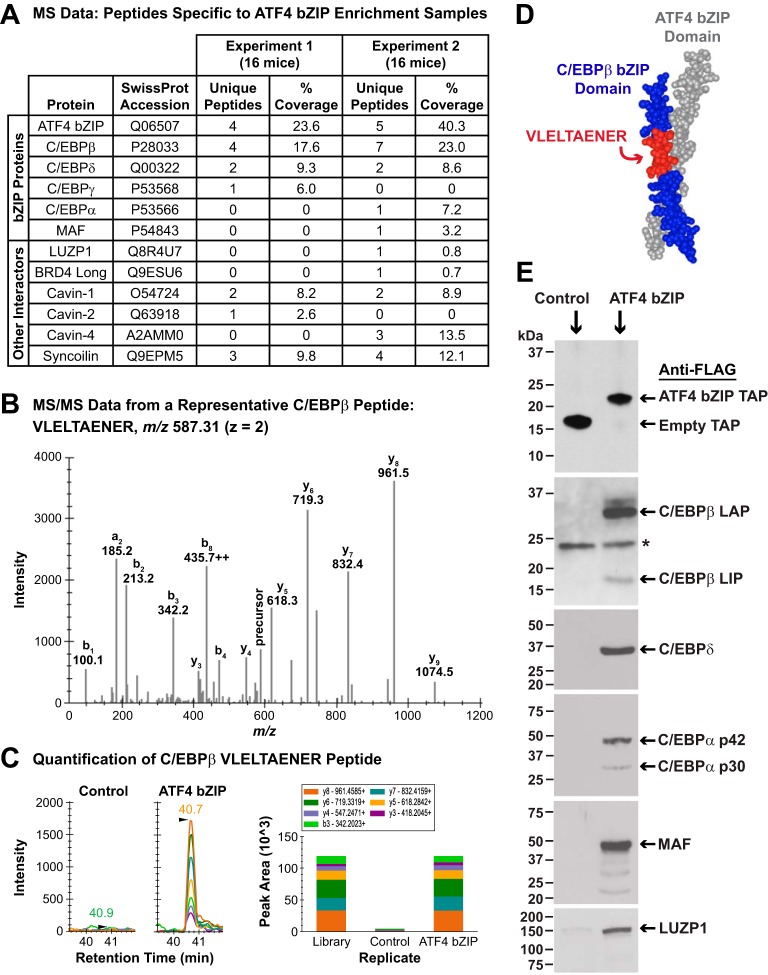
**Identification of proteins that interact with the ATF4 bZIP domain in mouse skeletal muscle fibers.** *A,* control and ATF4 bZIP pulldown samples from two independent, time-separated experiments were obtained as described in Fig. 1*D* and then subjected to MS and a data analysis workflow that is fully detailed under “Experimental procedures” and the supporting information. The *table* in *A* summarizes the mass spectrometric data for ATF4 and the high-confidence ATF4-interacting proteins that were identified in the ATF4 bZIP pulldown samples. For proteins with only one peptide identified, the corresponding MS/MS were manually inspected (also see under “Experimental procedures” and Fig. S2). *B,* tandem mass spectrum (MS/MS) from a representative C/EBPβ peptide VLELTAENER (SwissProt accession P28033) spanning residues 254–263 with the precursor ion at *m/z* 587.31 2^+^ identified in the ATF4 bZIP affinity-enrichment sample. *C, left*, quantification of C/EBPβ peptide VLELTAENER using DIA MS2-based quantification; *panels* show extracted ion chromatograms for the MS2 fragment ions in the control and ATF4 bZIP affinity enrichment sample. *Right*, MS/MS spectral library “simulation” indicating relative fragment ion distribution and calculated peak areas under the curve for the C/EBPβ peptide VLELTAENER in control and ATF4 bZIP affinity enrichment samples. *D,* mapping of VLELTAENER to the crystal structure of the ATF4 and C/EBPβ bZIP domains (19). *E,* TA muscle fibers of 12 mice were transfected with 20 μg of empty TAP plasmid (one TA per mouse) or 20 μg of ATF4 bZIP TAP plasmid (the contralateral TA in each mouse). Seven days post-transfection, bilateral TA muscles were harvested and used to prepare pooled protein extracts from each of the two groups of skeletal muscles (control and ATF4 bZIP). The pooled protein extracts were then subjected to pulldown with anti-FLAG magnetic beads, followed by SDS-PAGE and immunoblot analysis using HRP-conjugated mouse monoclonal anti-FLAG IgG (*upper panel*), mouse monoclonal anti-C/EBPβ IgG, rabbit polyclonal anti-C/EBPδ antibody, rabbit monoclonal anti-C/EBPα IgG, rabbit polyclonal anti-MAF antibody, and rabbit polyclonal anti-LUZP1 antibody, as indicated. *Asterisk* in the anti-C/EBPβ immunoblot denotes a nonspecific cross-reacting protein.

We subjected aliquots of the affinity enrichment samples to SDS-PAGE and silver staining, which revealed multiple proteins that were absent in the control sample and present in the ATF4 bZIP sample, including the ATF4 bZIP TAP construct (Fig. 1*E*). We then used mass spectrometry (MS) to identify the proteins in the affinity enrichment samples. Across the two experiments, we identified a total of 35 proteins in the control samples and 68 proteins in the ATF4 bZIP samples (Table S1). To differentiate *bona fide* ATF4-interacting proteins from nonspecific interactors, we subjected the MS data to an informatics workflow, defining high-confidence ATF4 interactors as proteins that 1) were detected in the ATF4 bZIP samples in at least one of the two independent pulldown experiments, 2) were not detected in control samples (empty TAP construct) in these or nine other independent experiments, and 3) did not interact with five other bait proteins that we have studied by similar methods, including Gadd45a (14). Altogether, we identified 12 proteins that met these criteria and thus appear to specifically interact, directly or indirectly, with ATF4 in skeletal muscle fibers *in vivo* (Fig. 2*A*).

As expected, the most abundant peptides in the ATF4 bZIP affinity enrichment samples were from ATF4 bZIP domain (Fig. 2*A*). Interestingly, we did not detect any ATF4 peptides from outside the bZIP domain that would have indicated the presence of endogenous ATF4 and thus an ATF4 homodimer. Importantly, however, we identified five other bZIP proteins in the ATF4 bZIP pulldown samples: C/EBPβ, C/EBPδ, C/EBPα, C/EBPγ, and MAF/c-Maf (Fig. 2*A*). Previous studies have shown that ATF4 is capable of heterodimerizing with C/EBPβ, C/EBPδ, C/EBPα, C/EBPγ, and MAF (19–24, 27, 28, 39, 40); together, these five proteins represent ∼17% of the bZIP proteins that are known to bind ATF4 with higher affinities than ATF4 itself (20, 21). Because an individual bZIP domain can only interact with one other bZIP domain at a time, these data indicated the existence of at least five distinct ATF4 heterodimers in skeletal muscle fibers: ATF4–C/EBPβ, ATF4–C/EBPδ, ATF4–C/EBPα, ATF4–C/EBPγ, and ATF4–MAF.

All of the identified bZIP proteins are known to be of very low abundance in skeletal muscle. For example, in a recent deep proteomics study that estimated the relative abundance of proteins in total cellular lysates of mouse skeletal muscle, C/EBPβ and C/EBPγ each represented <0.0001% of total skeletal muscle protein, and the relative amounts of ATF4, C/EBPδ, C/EBPα, and MAF were below the limit of detection (41). In our ATF4 bZIP pulldown samples, we identified C/EBPβ and C/EBPδ in both of the two independently-performed experiments, and we identified C/EBPα, C/EBPγ, and MAF in one of the two experiments (Fig. 2*A*). Although potential differences in peptide ionization efficiencies make it difficult to definitively determine the relative abundance of these bZIP proteins, the number of observed peptides suggests that the ATF4–C/EBPβ may be the most abundant ATF4 heterodimer in skeletal muscle fibers, followed by ATF4–C/EBPδ > ATF4–C/EBPα ≈ ATF4–C/EBPγ ≈ ATF4–MAF.

Interestingly, one of the C/EBPβ peptides that we detected in the ATF4 bZIP affinity enrichment samples (VLELTAENER; Fig. 2, *B* and *C*) is present in the only available crystal structure of ATF4, which consists of an ATF4 bZIP domain complexed to a C/EBPβ bZIP domain (19). Fig. 2*D* illustrates how that portion of C/EBPβ (VLELTAENER) interacts with residues within the ATF4 bZIP domain. Raw MS data for other ATF4-interacting peptides may be found in the supporting information.

The *C/EBP*β gene generates a single mRNA transcript that can generate three protein isoforms via alternative translation initiation sites (42, 43). The three C/EBPβ isoforms are called LIP (liver-inhibitory protein), LAP (liver-activating protein), and LAP*. LIP and LAP are considered to be the major C/EBPβ isoforms (42). All three C/EBPβ isoforms contain a C-terminal bZIP domain, and their N-terminal extensions vary in length. LIP, the shortest isoform, lacks a transcription activation domain and acts as a dominant-negative transcriptional repressor due to its capacity to form inactive heterodimers with other bZIP proteins (42, 43). In contrast, LAP and LAP* both possess a transcription activation domain and are transcriptionally active (42, 43). In both of our pulldown experiments, we identified peptides that are present in the two long C/EBPβ isoforms (LAP and LAP*) but not in the short C/EBPβ isoform, LIP. We also identified peptides that are found in all three C/EBPβ isoforms, such as VLELTAENER (Fig. 2, *B* and *C*).

*C/EBP*α also has multiple protein isoforms (42, 43), and the single unique C/EBPα peptide that we identified is common to all C/EBPα isoforms. C/EBPδ, C/EBPγ, and MAF are each generally considered to exist as only one isoform. In addition to the five bZIP proteins, the ATF4 bZIP affinity enrichment samples also contained six proteins that do not contain bZIP domains. Those six proteins were leucine zipper protein 1 (LUZP1), the long isoform of bromodomain-containing protein 4 (Brd4), three members of the cavin family (cavin-1/PTRF, cavin-2/SDPR, and cavin-4/MURC), and syncoilin (Fig. 2*A*). To our knowledge, none of these proteins were previously known to interact with ATF4.

To further investigate these protein–protein interactions by a complementary method, we performed one-step FLAG pulldowns from mouse muscle lysates containing either the empty TAP construct or the ATF4 bZIP TAP construct, followed by immunoblot analyses of the pulldown samples. Although less sensitive than MS, immunoblot analysis confirmed the presence of C/EBPβ, C/EBPδ, C/EBPα, MAF, and LUZP1 in the ATF4 bZIP pulldown samples (Fig. 2*E*). For C/EBPβ, we observed the two major isoforms, LAP and LIP (42), with LAP appearing to be ∼10-fold more abundant than LIP by densitometry (Fig. 2*E*). We did not observe the minor C/EBPβ isoform LAP* (which migrates slightly above 37 kDa; see Fig. 2*E*); however, a portion of ATF4-associated LAP migrated in a way that suggested it may be post-translationally modified (Fig. 2*E*). For C/EBPα, we also observed the two major isoforms, p42 and p30 (42), with p42 being more abundant than p30 (Fig. 2*E*).

In these immunoblot analyses, we were unable to detect C/EBPγ or BRD4, perhaps due to their low abundance and/or limitations of the available antibodies against these proteins, and we did not investigate syncoilin or the cavins. Importantly, and consistent with the MS data, immunoblot analysis did not reveal any endogenous ATF4 in the ATF4 bZIP pulldown samples (data not shown). Thus, we were unable to detect any evidence of an ATF4 homodimer in skeletal muscle fibers by either immunoblot or MS analyses. However, both methods revealed multiple ATF4 heterodimers, with the most abundant detected heterodimer appearing to be ATF4–C/EBPβ(LAP).

### Of the ATF4 heterodimers identified in muscle fibers, only ATF4–C/EBPβ is required for ATF4-mediated skeletal muscle atrophy

**Figure 3.**
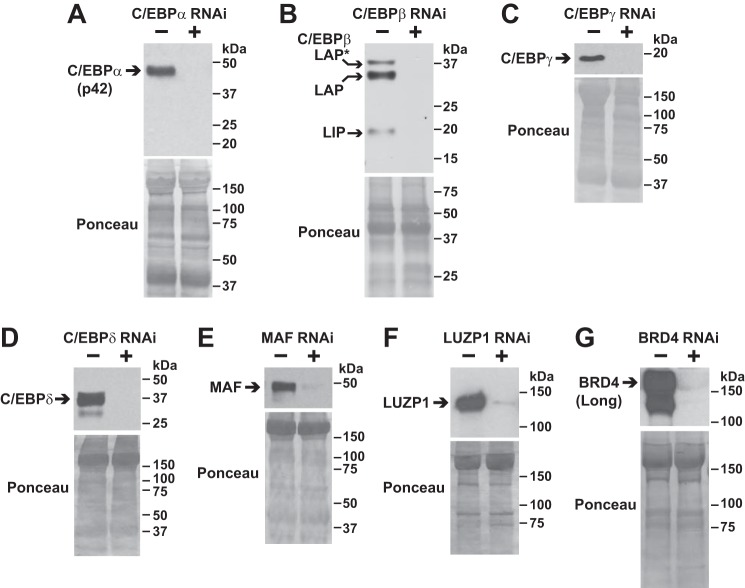
**Development of RNAi constructs that target ATF4 interactors in mouse skeletal muscle fibers.** Mouse TA muscles were co-transfected with 10 μg of cDNA (C/EBPα plasmid in *A*; C/EBPβ plasmid in *B*; C/EBPγ plasmid in *C*; C/EBPδ plasmid in *D*; MAF plasmid in *E*; LUZP1–FLAG plasmid in *F*; and BRD4–FLAG plasmid in *G*) plus either 20 μg of nontargeting control RNAi plasmid or 20 μg of RNAi plasmid targeting C/EBPα (*A*), C/EBPβ (*B*), C/EBPγ (*C*), C/EBPδ (*D*), MAF (*E*), LUZP1 (*F*), or BRD4 (*G*), as indicated. Seven days post-transfection, bilateral TA muscles were harvested for SDS-PAGE and immunoblot analysis using rabbit monoclonal anti-C/EBPα IgG (*A*), mouse monoclonal anti-C/EBPβ IgG (*B*), rabbit polyclonal anti-C/EBPγ antibody (*C*), rabbit polyclonal anti-C/EBPδ antibody (*D*), rabbit polyclonal anti-MAF antibody (*E*), and HRP-conjugated mouse monoclonal anti-FLAG IgG (*F* and *G*). Membranes were stained with Ponceau S to confirm equal loading.

To test the hypothesis that one or more ATF4-interacting proteins might be required for ATF4-mediated skeletal muscle atrophy, we performed an *in vivo*, RNAi-based phenotypic screen of the five identified bZIP proteins (C/EBPα, C/EBPβ, C/EBPγ, C/EBPδ, and MAF). In this screen, we also tested LUZP1 and the long isoform of BRD4 due to their potential roles in transcriptional regulation (44, 45). As a first step, we developed RNA interference (RNAi) constructs that target the mRNAs encoding C/EBPα, C/EBPβ, C/EBPγ, C/EBPδ, MAF, LUZP1, and the long isoform of BRD4, leading to specific knockdown of these proteins in mouse skeletal muscle *in vivo* (Fig. 3, *A–G*). We then co-transfected skeletal muscle fibers of young healthy mice with plasmid encoding ATF4 plus plasmid encoding either a nontargeting RNAi control construct (in one TA of each animal) or RNAi constructs that target C/EBPα, C/EBPβ, C/EBPγ, C/EBPδ, MAF, BRD4, or LUZP1 (in the contralateral TA of each animal).

**Figure 4.**
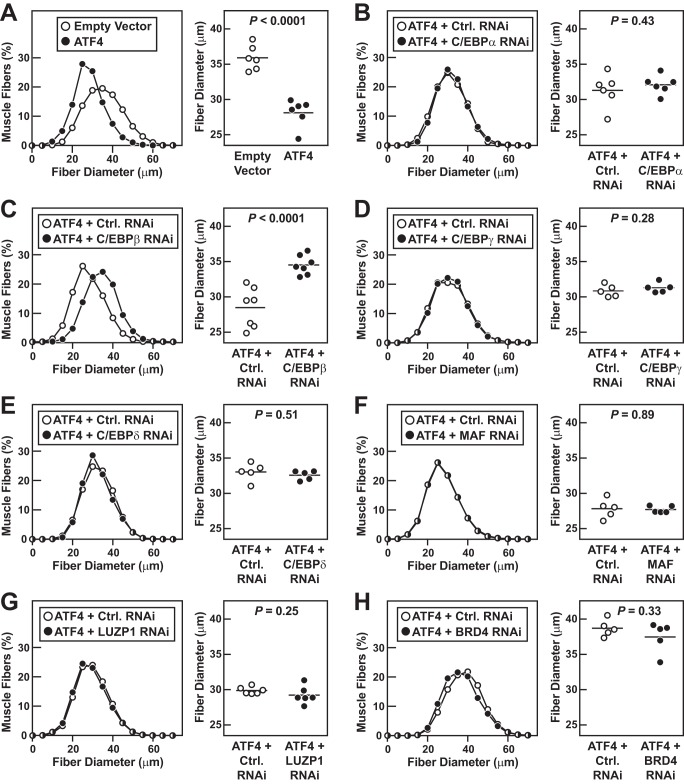
***In vivo* RNAi-based phenotypic screen of ATF4 interactors in mouse skeletal muscle fibers.** *A,* one TA muscle per mouse was transfected with 5 μg of empty pcDNA plasmid plus 2 μg of eGFP plasmid, and the contralateral TA in each mouse was transfected with 5 μg of ATF4–FLAG plasmid plus 2 μg of eGFP plasmid. *B–H*, one TA per mouse was transfected with 5 μg of ATF4–FLAG plasmid plus 10 μg of control RNAi plasmid, and the contralateral TA in each mouse was transfected with 5 μg of ATF4–FLAG plasmid plus 10 μg of RNAi plasmid targeting C/EBPα (*B*), C/EBPβ (*C*), C/EBPγ (*D*), C/EBPδ (*E*), MAF (*F*), LUZP1 (*G*), or BRD4 (*H*), as indicated. *A–H*, 7 days post-transfection, bilateral TAs were harvested for histological analysis. *Left panels*: size distribution of >4700 transfected muscle fibers from ≥5 muscles per condition. *Right panels*: each data point represents the mean fiber diameter from one TA (>550 fibers measured per muscle), and *horizontal bars* denote average of the means. *p* values were determined with paired *t* tests.

**Figure 5.**
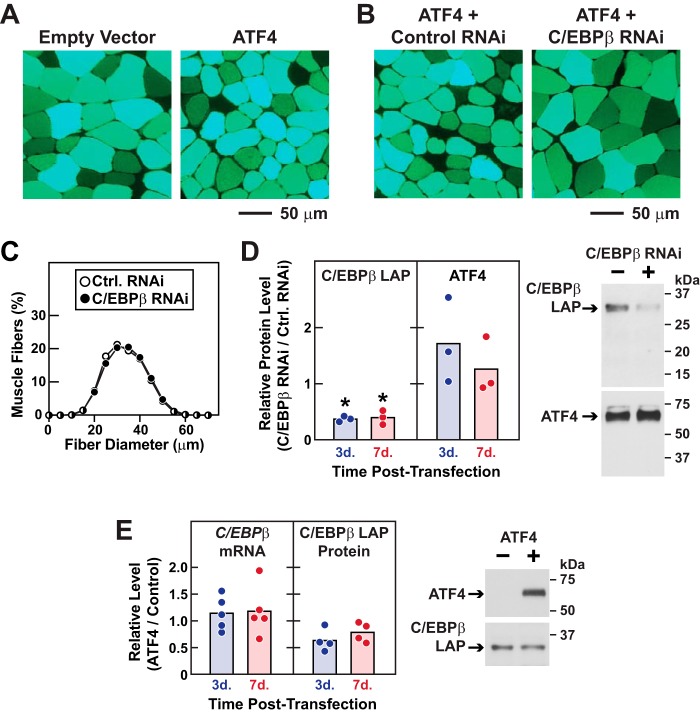
**C/EBPβ is required for ATF4-mediated muscle atrophy.** *A,* representative fluorescence microscopy images of muscle cross-sections from Fig. 4*A. B,* representative fluorescence microscopy images of muscle cross-sections from Fig. 4*C. C,* one TA muscle per mouse was transfected with 10 μg of control RNAi plasmid, and the contralateral TA in each mouse was transfected with 10 μg of C/EBPβ RNAi plasmid, as indicated. Seven days post-transfection, bilateral TAs were harvested for histological analysis. Data are size distributions of >7000 transfected muscle fibers from six muscles per condition. *D,* one TA per mouse was transfected with 5 μg of ATF4–FLAG plasmid plus 10 μg of control RNAi plasmid, and the contralateral TA in each mouse was transfected with 5 μg of ATF4–FLAG plasmid plus 10 μg of C/EBPβ RNAi plasmid. Bilateral TAs were harvested 3 and 7 days post-transfection for immunoblot analyses using mouse monoclonal anti-C/EBPβ IgG and HRP-conjugated mouse monoclonal anti-FLAG IgG for ATF4. *Left,* quantification of C/EBPβ and ATF4 protein. In each mouse, the level in the C/EBPβ RNAi-transfected TA was normalized to the level in the contralateral (control RNAi) TA. Each *circle* represents one mouse, and *horizontal bars* denote the means. *Stars* denote significant knockdown by C/EBPβ RNAi relative to control RNAi in the same animal by paired *t* test (*p* < 0.05). *Right*, representative immunoblots of total muscle lysates harvested 3 days post-transfection. *E,* one TA muscle per mouse was transfected with 5 μg of empty pcDNA3 plasmid (control), and the contralateral TA in each mouse was transfected with 5 μg of ATF4–FLAG plasmid. Bilateral TAs were harvested 3 and 7 days post-transfection for qPCR and immunoblot analyses. *Left,* quantification of *C/EBP*β mRNA and C/EBPβ protein. In each mouse, the level in the ATF4-transfected TA was normalized to the level in the contralateral (control) TA. Each *circle* represents one mouse, and *horizontal bars* denote the means. *Right,* representative immunoblots of total muscle lysates harvested 7 days post-transfection.

Fig. 4*A* shows that forced ATF4 expression induces muscle fiber atrophy, similar to what has been previously reported (5). RNAi-mediated knockdown of C/EBPα, C/EBPγ, C/EBPδ, MAF, BRD4, and LUZP1 did not alter muscle fiber size in the presence of ATF4 (Fig. 4, *B* and *D–H*). However, RNAi-mediated knockdown of C/EBPβ significantly inhibited the effect of ATF4 on muscle fiber size (Fig. 4*C*). This is further illustrated by the photomicrographs in Fig. 5, *A* and *B*; ATF4 alone induces muscle fiber atrophy (Fig. 5*A*), and under those conditions the C/EBPβ RNAi construct prevents muscle fiber atrophy (Fig. 5*B*). Thus, among the seven tested RNAi constructs, only C/EBPβ RNAi reduced ATF4-mediated muscle atrophy.

Importantly, the C/EBPβ RNAi construct had no effect on muscle fiber size in the absence of ATF4 (Fig. 5*C*), indicating that C/EBPβ is required for induction of muscle fiber atrophy (as opposed to suppression of muscle fiber hypertrophy). Moreover, C/EBPβ RNAi did not reduce ATF4 expression, but it reduced C/EBPβ, as expected (Fig. 5*D*). Because ATF4 activates the *C/EBP*β gene in some systems (29), we tested whether ATF4 might increase C/EBPβ expression in skeletal muscle fibers. However, we found that ATF4 did not increase *C/EBP*β mRNA or C/EBPβ protein in muscle fibers (Fig. 5*E*), consistent with the well-established fact that ATF4 target genes are highly context-dependent. These findings, coupled with the protein–protein interaction data in Fig. 2, strongly suggest that ATF4 promotes muscle atrophy by forming a heterodimer with C/EBPβ.

**Figure 6.**
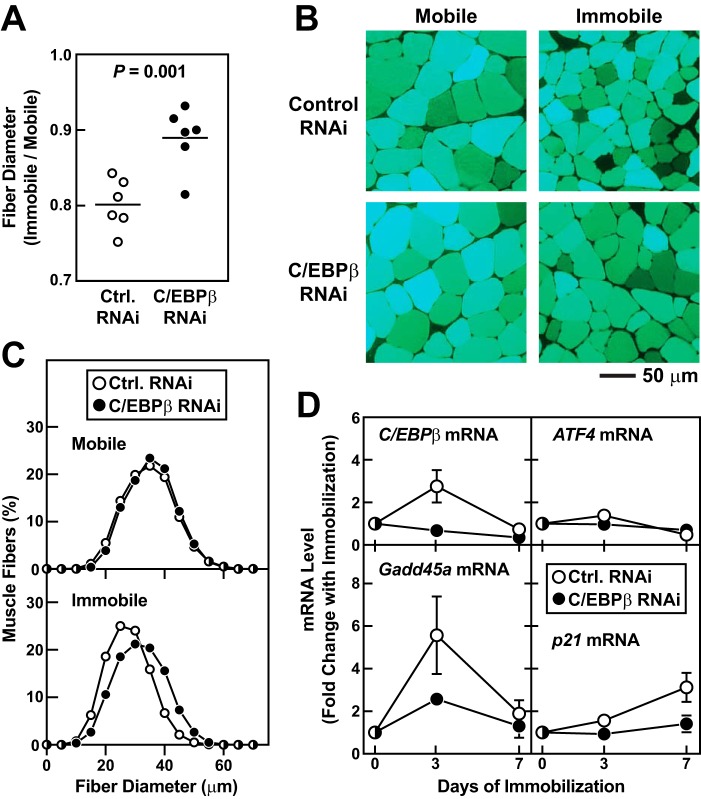
**C/EBPβ is required for immobilization-induced *Gadd45a* expression and muscle atrophy.** *A–C,* in one cohort of mice, bilateral TAs were transfected with 20 μg of control RNAi plasmid. In a second cohort of mice, bilateral TAs were transfected with 20 μg of C/EBPβ RNAi plasmid. Three days after transfection, one hind limb in each mouse was immobilized, and 7 days later (10 days post-transfection), bilateral TAs were harvested for histological analysis. *A,* in each mouse, the average muscle fiber diameter in the immobilized TA was normalized to the average muscle fiber diameter in the contralateral control (mobile) TA. Each *data point* represents the value from one mouse, and the *horizontal bars* denote means. *p* value was determined with an unpaired *t* test. *B,* representative fluorescence microscopy images of muscle cross-sections. *C,* size distribution of all muscle fibers from *A*; each distribution represents >6800 muscle fibers from six muscles. *D,* TA muscles were transfected and immobilized as in *A–C* and then harvested at the indicated days post-immobilization for qPCR analysis of *C/EBP*β, *ATF4*, *Gadd45a,* and *p21* mRNAs. mRNA levels in immobilized muscles were normalized to the average level in control RNAi-transfected mobile muscles. Data are means ± S.D. from four to six mice per condition. Some *error bars* are too small to see. Two-way ANOVA revealed a significant effect of time on all four mRNAs, and a significant effect of C/EBPβ RNAi on *C/EBP*β mRNA (*p* < 0.0001), *Gadd45a* mRNA (*p* < 0.001), and *p21* mRNA (*p* < 0.0001) but not *ATF4* mRNA.

To test the hypothesis that C/EBPβ might be required for a natural ATF4-dependent form of skeletal muscle atrophy, we investigated the potential role of C/EBPβ in immobilization-induced muscle atrophy. Immobilization promotes muscle fiber atrophy by stimulating ATF4-dependent expression of *Gadd45a* in skeletal muscle fibers (2). To determine whether C/EBPβ might also be required for immobilization-induced *Gadd45a* expression and muscle atrophy, we transfected mouse TA muscle fibers *in vivo* with plasmids encoding either nontargeting control RNAi or C/EBPβ RNAi, and then immobilized the transfected muscles for 7 days. As expected, under control conditions, immobilization caused muscle fiber atrophy (Fig. 6, *A–C*) and increased the level of *Gadd45a* mRNA (Fig. 6*D*). Immobilization also increased the level of another important ATF4-dependent mRNA, *p21* (Fig. 6*D*) (1). In addition, immobilization transiently increased the level of *C/EBP*β mRNA (Fig. 6*D*). C/EBPβ RNAi significantly reduced *C/EBP*β mRNA in immobilized muscles, without affecting the level of *ATF4* mRNA (Fig. 6*D*). Furthermore, C/EBPβ RNAi significantly decreased immobilization-induced muscle fiber atrophy (Fig. 6, *A–C*) and *Gadd45a* and *p21* expression (Fig. 6*D*). These data indicate that C/EBPβ plays an essential role in skeletal muscle atrophy caused by at least one common, naturally occurring ATF4-dependent stress condition.

### ATF4–C/EBPβ heterodimer activates the Gadd45a gene in skeletal muscle fibers via an evolutionarily conserved ATF–C/EBP composite site

Because immobilization increases *Gadd45a* mRNA in an ATF4-dependent manner (2) and in a C/EBPβ-dependent manner (Fig. 6*D*), we hypothesized that the *Gadd45a* gene might contain a regulatory element that is activated by the ATF4–C/EBPβ heterodimer. As an initial test of this hypothesis, we generated luciferase reporter genes containing portions of the promoter region of the mouse *Gadd45a* gene (ranging between −2637 and +163 bp relative to the transcription start site). We then tested whether those reporter genes might be activated by immobilization and/or ATF4 overexpression in mouse skeletal muscle fibers *in vivo*. However, we found that neither immobilization nor ATF4 overexpression activated the *Gadd45a* promoter in skeletal muscle fibers. This led us to look elsewhere for the putative regulatory element.

**Figure 7.**
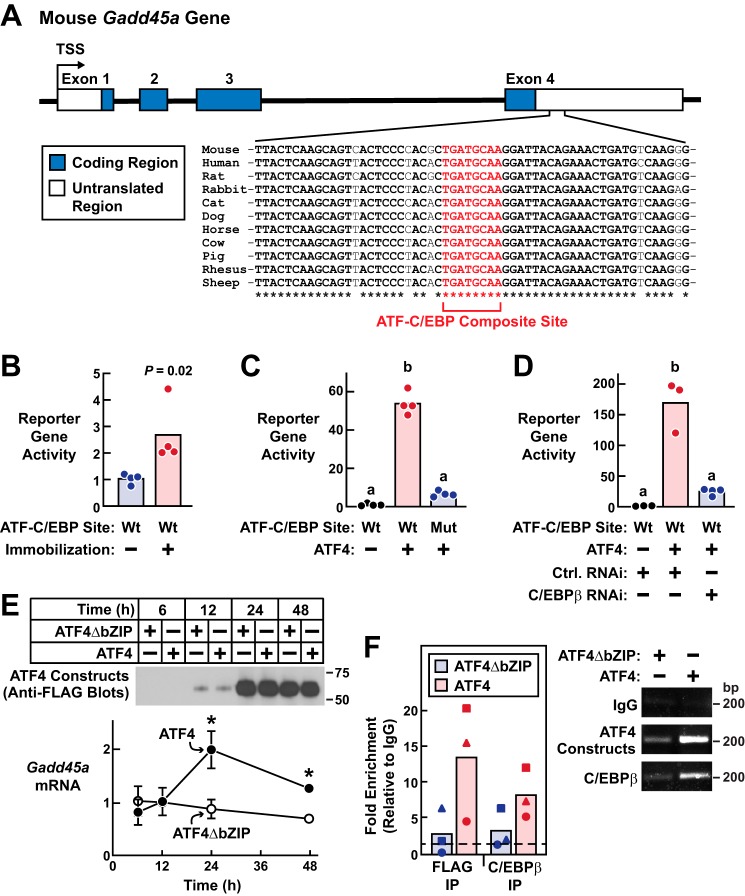
**ATF4–C/EBPβ binds and activates an evolutionarily-conserved regulatory element in the *Gadd45a* gene.** *A,* schematic illustration of the mouse *Gadd45a* gene and the conserved ATF–C/EBP composite site in the 3′-untranslated region (UTR) of exon 4. To generate the “WT” reporter plasmid described in *B–D*, we placed this ATF–C/EBP composite site plus some flanking sequence from the 3′-UTR (+1733 to +1933 bp relative to the *Gadd45a* transcription start site) upstream of the transcription start site and luciferase-coding region in the pGL3-Basic reporter plasmid. To generate the “mutant” reporter plasmid described in *C*, we changed the ATF–C/EBP composite site in the WT reporter from TGATGCAA to CAGAATGG. *B,* bilateral mouse TA muscles were transfected with 5 μg of WT reporter plasmid plus 0.3 μg of *Renilla* plasmid. Three days after transfection, one hind limb in each mouse was immobilized. Six days post-transfection, bilateral TAs were harvested for analysis of reporter gene activity. In each muscle, the level of reporter gene activity was normalized to the average level in the transfected mobile TAs. Each *circle* represents one transfected muscle, and *bars* denote the means. *p* value was determined with a paired *t* test. *C,* mouse TA muscles were transfected with 5 μg of WT reporter plasmid + 5 μg of empty plasmid (pcDNA3) + 0.3 μg of *Renilla* plasmid (group 1); 5 μg of WT reporter plasmid + 5 μg of ATF4–FLAG plasmid + 0.3 μg of *Renilla* plasmid (group 2); or 5 μg of mutant reporter plasmid + 5 μg of ATF4–FLAG plasmid + 0.3 μg of *Renilla* plasmid (group 3), as indicated. TA muscles were harvested 7 days post-transfection for analysis of reporter gene activity, which was then normalized to the average level in group 1. Each *circle* represents one transfected muscle, and *bars* denote the means. *Different letters* denote statistically differences (*p* ≤ 0.05) by one-way ANOVA with Dunnett's post test. *D,* mouse TA muscles were transfected with 5 μg of WT reporter plasmid + 5 μg of pcDNA3 + 10 μg of control RNAi plasmid + 0.3 μg of *Renilla* plasmid (group 1); 5 μg of WT reporter plasmid + 5 μg of ATF4–FLAG plasmid + 10 μg of control RNAi plasmid + 0.3 μg of *Renilla* plasmid (group 2); or 5 μg of WT reporter plasmid + 5 μg of ATF4–FLAG plasmid + 10 μg of C/EBPβ RNAi plasmid + 0.3 μg of *Renilla* plasmid (group 3). TA muscles were harvested 7 days post-transfection for analysis of reporter gene activity, which was then normalized to the average level in group 1. Each *circle* represents one transfected muscle, and *bars* denote the means. *Different letters* denote statistically differences (*p* ≤ 0.05) by one-way ANOVA with Dunnett's post test. *E,* fully differentiated C2C12 myotubes were treated with recombinant adenoviruses expressing either ATF4–FLAG or a full-length transcriptionally inactive ATF4–FLAG construct (ATF4ΔbZIP) and then harvested at the indicated times post-adenovirus treatment for immunoblot analysis using HRP-conjugated mouse monoclonal anti-FLAG IgG (*top*) and qPCR analysis of *Gadd45a* mRNA (*bottom*). In the qPCR analysis, data points are means ± S.D. from three replicates/condition. Some *error bars* are too small to see. *, *p* ≤ 0.05 relative to ATF4ΔbZIP. *F,* fully-differentiated C2C12 myotubes were treated as in *E* and harvested 24 h after adenovirus treatment. ChIP was then performed using either control anti-IgG, anti-FLAG, or anti-C/EBPβ, followed by qPCR analysis of the same exon 4 *Gadd45a* sequence used in the WT reporter plasmid described in *A–D, left*, results from three independent experiments. Each *circle*, *triangle,* or *square* represents data from one independent experiment; data from the same experiment were assigned the same symbols. Relative to IgG, fold enrichment was significantly increased in the ATF4 but not ATF4ΔbZIP samples (*p* < 0.05 by one-way ANOVA with Dunnett's post test). *Right,* representative agarose gel images.

The schematic in Fig. 7*A* illustrates the mouse *Gadd45a* gene, which contains four exons. In the 3′-untranslated region (UTR) of exon 4, we identified a nonpalindromic sequence (TGATGCAA) that is identical to ATF–C/EBP composite sites that are activated by ATF4–C/EBP heterodimers in several other genes (Fig. 7*A*) (29). Moreover, the ATF–C/EBP composite site in *Gadd45a* exon 4 as well as much of its surrounding sequence are strongly conserved in mice, humans, and other available mammalian genomes (Fig. 7*A*). We therefore inserted this portion of the 3′-UTR of *Gadd45a* exon 4 (+1733 to +1933 relative to the transcription start site) into the pGL3-Basic luciferase reporter plasmid (upstream of the transcription start site and luciferase-coding region), transfected that reporter gene into mouse TA muscle fibers, and then tested the hypothesis that 3 days of immobilization might stimulate reporter gene activity. Importantly, we found that immobilization significantly increased the activity of this reporter gene in mouse skeletal muscle fibers *in vivo* (Fig. 7*B*). Furthermore, this regulatory element appeared to be autoregulatory because it was induced by a stimulus (3 days of immobilization) that increases *Gadd45a* mRNA (Fig. 6*D*) (2, 3) but not any of the mRNAs that arise from evolutionarily-conserved genes near the *Gadd45a* gene (*e.g. Gng12*, *Serbp1,* and *Il12rb2*, as assessed by genome-wide mRNA expression arrays (3) and qPCR (data not shown)). Thus, exon 4 of the *Gadd45a* gene contains an evolutionarily-conserved, immobilization-inducible response element with a consensus ATF–C/EBP composite site.

To test the hypothesis that ATF4 might activate the response element in the *Gadd45a* gene, we co-transfected non-immobilized TA muscle fibers with the same reporter gene plus plasmid encoding ATF4. We found that ATF4 strongly activated the reporter gene in mouse skeletal muscle fibers *in vivo* (Fig. 7*C*). Furthermore, the capacity of ATF4 to activate the reporter gene in muscle fibers was almost completely abolished by site-directed mutagenesis of the ATF–C/EBP composite site (from TGATGCAA to CAGAATGG; Fig. 7*C*) and by RNAi-mediated knockdown of C/EBPβ (Fig. 7*D*). Mutagenesis of the ATF–C/EBP composite site also abolished immobilization-induced reporter gene activity (Fig. S3).

To determine whether ATF4 and C/EBPβ might interact with the response element in the *Gadd45a* gene, we performed ChIP in C2C12 skeletal myotubes, an *in vitro* model of skeletal muscle. Fully-differentiated myotubes were treated with recombinant adenovirus encoding either ATF4 or a full-length, transcriptionally-inactive ATF4 construct in which the bZIP domain has been mutated to reduce DNA binding (ATF4ΔbZIP (2, 4)). In this system, ATF4 but not ATF4ΔbZIP induces *Gadd45a* expression and myotube atrophy (2), similar to their effects *in vivo*. As expected, both ATF4 and ATF4ΔbZIP were highly expressed, but only ATF4 increased *Gadd45a* mRNA (Fig. 7*E*). Furthermore, the induction of *Gadd45a* mRNA was associated with the presence of both ATF4 and C/EBPβ at the response element in the *Gadd45a* gene (Fig. 7*F*). Taken together, these data strongly suggest that the ATF4–C/EBPβ heterodimer promotes skeletal muscle atrophy by directly activating the *Gadd45a* gene.

## Discussion

In this study, we sought to better understand how ATF4 promotes skeletal muscle atrophy. Based on elegant work from other systems (15–31, 46, 47), we hypothesized that ATF4 may promote muscle atrophy by heterodimerizing with another bZIP family member. To test that hypothesis, we used a biochemical approach to search for ATF4 heterodimerization partners in mouse skeletal muscle fibers *in vivo*. We then performed functional investigations of the identified proteins in mouse skeletal muscle and cultured skeletal myotubes.

**Figure 8.**
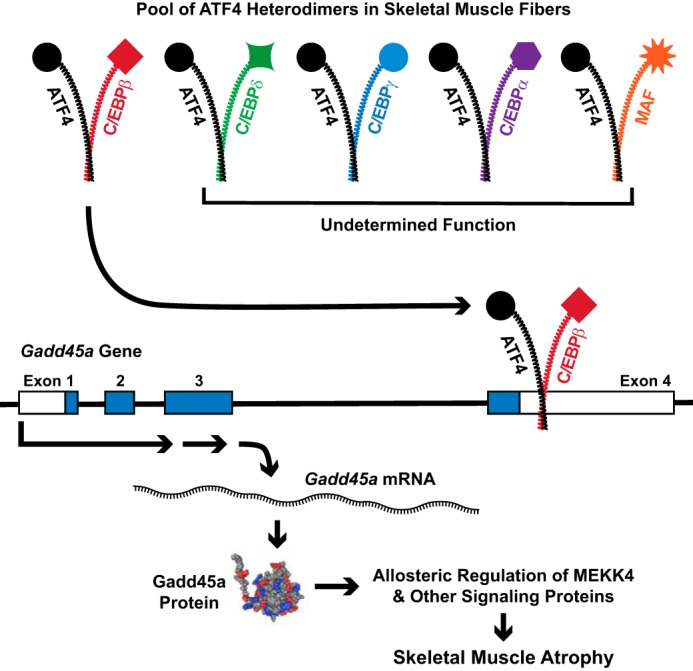
**Proposed model illustrating the identified pool of ATF4 heterodimers in skeletal muscle fibers and the role of the ATF4–C/EBPβ heterodimer in skeletal muscle atrophy.** The Gadd45a NMR structure and the downstream effects of Gadd45a in skeletal muscle are taken from Refs. 48 and 1, respectively.

The results of this study, coupled with previous findings, suggest a model that is illustrated in Fig. 8. In skeletal muscle fibers, ATF4 forms at least five distinct heterodimeric bZIP transcription factors. Moreover, one of these heterodimers, ATF4–C/EBPβ, is required for skeletal muscle atrophy. The ATF4–C/EBPβ heterodimer promotes skeletal muscle atrophy by interacting with an evolutionarily conserved ATF–C/EBP composite site in the 3′-UTR of exon 4 of the *Gadd45a* gene. The three-way interaction between ATF4, C/EBPβ, and the ATF–C/EBP composite site activates the *Gadd45a* gene. This, in turn, increases the level of Gadd45a, an 18-kDa globular protein (48), that promotes skeletal muscle atrophy by allosterically activating the protein kinase MEKK4 (14). Gadd45a also interacts with 10 other protein kinases and three protein-tyrosine phosphatases in skeletal muscle fibers, and those interactions may also contribute to muscle atrophy (1, 14).

The finding of multiple ATF4 heterodimers within the same cell type *in vivo* is, to our knowledge, novel. Certainly in skeletal muscle fibers, ATF4 should no longer be considered to be a single transcription factor, but rather an essential component of five or more transcriptional regulatory complexes. This concept of multiple co-existing ATF4 heterodimers may also hold true in other cell types and might help to resolve some existing and future questions regarding the biological effects of ATF4, which are often complex and highly context-dependent even in the same cell type. By combining two different bZIP proteins, each heterodimer potentially acquires its own specific regulation, target genes, and biological effects.

In skeletal muscle fibers, the functions of the ATF4–C/EBPδ, ATF4–C/EBPα, ATF4–C/EBPγ, and ATF4–MAF heterodimers are not yet known. It seems likely that each heterodimer may regulate its own specific subset of ATF4-dependent mRNAs in skeletal muscle, but further studies will be required to test that hypothesis. With respect to C/EBPβ, our data indicate that ATF4 heterodimerizes with two different C/EBPβ isoforms in muscle fibers, LAP and LIP. The ATF4–C/EBPβ(LAP) heterodimer is transcriptionally active (42, 43) and appears to be the most abundant ATF4 heterodimer in skeletal muscle fibers. In contrast, the ATF4–C/EBPβ(LIP) heterodimer is much less abundant and predicted to be a transcriptional repressor, unable to activate target genes such as *Gadd45a* (42, 43). Thus, the available evidence strongly suggests that the relevant C/EBPβ isoform in skeletal muscle atrophy is LAP not LIP.

The newly-identified role of the ATF4–C/EBPβ heterodimer in muscle atrophy is based on evidence from two distinct models of skeletal muscle atrophy, limb immobilization and ATF4 overexpression. Going forward, it will be interesting to determine whether the ATF4–C/EBPβ heterodimer participates in other forms of skeletal muscle atrophy that were not investigated here, including age-related muscle atrophy and fasting-induced muscle atrophy (which require ATF4 (2, 4, 5)) and cancer-induced muscle atrophy (which requires C/EBPβ (49, 50)). It will also be important to determine whether the ATF4–C/EBPβ heterodimer is required for muscle atrophy caused by longer periods of immobilization than were tested here. Furthermore, it seems likely that the ATF4–C/EBPβ heterodimer may have other important target genes in skeletal muscle fibers, besides *Gadd45a*. For example, we speculate that the ATF4–C/EBPβ heterodimer may also target the *p21* gene, based on the findings that both ATF4 and C/EBPβ are required for the induction of *p21* mRNA during limb immobilization.

Along these same lines, it will also be interesting to determine whether the newly identified ATF4–C/EBPβ response element in the *Gadd45a* gene plays an important role in other conditions that cause muscle atrophy. Moreover, the high degree of evolutionary conservation of this ATF4–C/EBPβ response element is striking and suggests that it may be operative in other mammalian species, including humans and production livestock. In skeletal muscle, *Gadd45a* mRNA is highly induced by a wide range of muscle atrophy stimuli in mice, humans, and other mammals (2, 3, 5–12, 51), but the regulatory element that drives skeletal muscle *Gadd45a* expression has been unknown. Genetic experiments to investigate these questions are now underway.

Another interesting aspect of the regulatory element in the *Gadd45a* gene is that it resides within an exon. Exonic regulatory elements are abundant in mammalian genomes, and some autoregulate the genes in which they reside, whereas others regulate nearby genes (52). The regulatory element in the *Gadd45a* gene appears to be autoregulatory, inasmuch as its activity was associated with induction of *Gadd45a* mRNA but not mRNAs from nearby genes. Interestingly, in cultured mouse embryonic fibroblasts exposed to arsenite, leucine deprivation, or MG132, a different ATF4-binding site appears to mediate ATF4-dependent *Gadd45a* gene expression (47); however, that ATF4-binding site (which is located in the promoter region of the mouse *Gadd45a* gene, −2546 bp relative to the transcription start site) differs from an ATF–C/EBP composite site and is not conserved in humans, and we have not yet been able to find evidence that it is operative in mouse skeletal muscle.

This study also identified six novel ATF4-interacting proteins: LUZP1; the long isoform of BRD4; cavin-1; cavin-2; cavin-4; and syncoilin. These proteins do not contain bZIP domains, and we do not yet know whether they interact directly with ATF4 or indirectly via interactions with one or more of ATF4's bZIP heterodimerization partners. Interestingly, LUZP1, cavin-1, and cavin-2 contain leucine zippers (53, 54) that could potentially support interactions with the bZIP domain of ATF4, and BRD4 has been found to interact with C/EBPα and C/EBPβ (45). To our knowledge, LUZP1, the cavins, and syncoilin have no prior links to skeletal muscle atrophy; however, a recent study suggests that BRD4 activity in skeletal muscle may contribute to cancer-induced skeletal muscle atrophy (55). In this study, we found that LUZP1 and BRD4 are not required for muscle fiber atrophy caused by ATF4 overexpression, but further studies will be needed to determine whether these proteins play important roles in other models of muscle atrophy that involve ATF4. We have not yet performed functional studies of cavin-1, cavin-2, cavin-4, and syncoilin in skeletal muscle, but they too may be interesting topics for future investigation.

A potential limitation of this study is that technical aspects of our protocol may have caused us to miss other important proteins that interact with the ATF4 in skeletal muscle, including ATF4 itself. Although we found no evidence of an ATF4 homodimer in skeletal muscle fibers (consistent with previous data that the ATF4 leucine zipper is structurally unsuited to homodimerization (19)), we cannot rule out the existence of an ATF4 homodimer, especially since full-length ATF4 is highly unstable. Another potential limitation of this study is that although C/EBPβ emerged from our RNAi-based functional screen of ATF4-interacting proteins, we can envision several ways that the screen could generate false-negative results (*e.g.* insufficient knockdown of endogenous target proteins by the RNAi reagents used in the screen); thus, we cannot rule out the possibility that other ATF4 heterodimers may also play important roles in skeletal muscle atrophy. Further studies will be needed to resolve these questions.

In summary, this study identifies the ATF4–C/EBPβ heterodimer as an important mediator of skeletal muscle atrophy. These results provide new insight into the way that skeletal muscle atrophy occurs at the molecular level.

## Experimental procedures

### Chemicals

3X-FLAG peptide was from Sigma (catalogue no. F4799). HPLC-grade acetonitrile and water were obtained from Burdick & Jackson (Muskegon, MI). Reagents for protein chemistry (iodoacetamide, dithiothreitol (DTT), ammonium bicarbonate, formic acid (FA), and urea) were purchased from Sigma. Sequencing-grade trypsin was purchased from Promega (Madison, WI). HLB Oasis SPE cartridges were purchased from Waters.

### Buffers

Buffer A was 50 mm HEPES, pH 7.0, 150 mm NaCl, 5 mm MgCl_2_, 2 mm EGTA, 0.5% (v/v) Triton X-100, and 0.5 mm DTT. Buffer B was 50 mm HEPES, pH 7.0, 150 mm NaCl, 5 mm MgCl_2_, 2 mm EGTA, 0.1% (v/v) Triton X-100, and 0.5 mm DTT. Buffer C was 50 mm HEPES, pH 7.0, 150 mm NaCl, 5 mm MgCl_2_, 2 mm EGTA, 0.1% Triton X-100, 1% SDS, and 0.5 mm DTT. All buffers were made with ultrapure water (Gibco). Buffer D was 50 mm HEPES, pH 7.0, 4 mm EGTA, 10 mm EDTA, 1% (v/v) Triton X-100, 15 mm sodium pyrophosphate, 100 mm β-glycerophosphate, and 25 mm sodium fluoride. Immediately before use, Buffers A–D were supplemented with complete mini-EDTA–free protease inhibitor (Roche Applied Science) and PhosStop phosphatase inhibitor (Roche Applied Science) according to the manufacturer's instructions. Buffer E was 250 mm Tris·HCl, pH 6.8, 10% SDS, 25% glycerol, 0.2% (w/v) bromphenol blue, and 5% (w/v) 2-mercaptoethanol.

### Antibodies

Anti-FLAG M2 magnetic beads were from Sigma (catalogue no. M8823). S-Protein–agarose was from EMD Millipore (catalogue no. 69704). Horseradish peroxidase (HRP)-conjugated mouse monoclonal anti-FLAG IgG (catalogue no. A8592) and mouse monoclonal anti-FLAG IgG (catalogue no. F1804) were from Sigma. Mouse monoclonal anti-C/EBPβ IgG was from BioLegend (catalogue no. 606202). Rabbit monoclonal anti-C/EBPα IgG (catalogue no. 8178), rabbit polyclonal anti-C/EBPδ antibody (catalogue no. 2318), HRP-conjugated anti-mouse IgG (catalogue no. 7076), and HRP-conjugated anti-rabbit IgG (catalogue no. 7074) were from Cell Signaling Technology. Rabbit polyclonal anti-C/EBPγ antibody (catalogue no. ab74045) and rabbit polyclonal anti-MAF antibody (catalogue no. ab77071) were from Abcam. Rabbit polyclonal anti-LUZP1 antibody was from Proteintech (catalogue no. 17483-1-AP).

### Plasmids and recombinant adenoviruses

The empty TAP plasmid was described previously (56). The ATF4 bZIP TAP plasmid was generated by PCR-amplifying the bZIP domain (residues 278–349) from the ATF4–FLAG plasmid and then cloning that fragment into the empty TAP plasmid. The ATF4–FLAG plasmid has been described previously (5) and encodes full-length mouse ATF4 with three copies of the FLAG epitope tag at the N terminus, under control of the cytomegalovirus (CMV) promoter. The eGFP plasmid encodes eGFP under control of the CMV promoter. The control RNAi plasmid was described previously (2) and encodes emerald GFP (EmGFP) and a nontargeting pre-miRNA under the bicistronic control of the CMV promoter in the pcDNA6.2GW/EmGFP-miR plasmid (Invitrogen). The C/EBPα RNAi plasmid, C/EBPβ RNAi plasmid, C/EBPγ RNAi plasmid, C/EBPδ RNAi plasmid, MAF RNAi plasmid, LUZP1 RNAi plasmid, and BRD4 RNAi plasmid encode EmGFP and artificial pre-miRNAs targeting mouse C/EBPα, C/EBPβ, C/EBPγ, C/EBPδ, MAF, LUZP1, and BRD4, respectively, under bicistronic control of the CMV promoter; they were generated by ligating oligonucleotide duplexes (Invitrogen) targeting C/EBPα (Mmi506319), C/EBPβ (NM_009883_158), C/EBPγ (Mmi506328), C/EBPδ (Mmi506324), MAF (Mmi514115), LUZP1 (Mmi539662), and BRD4 (Mmi552649), respectively, into the pcDNA6.2GW/EmGFP-miR plasmid. The C/EBPα plasmid, C/EBPβ plasmid, C/EBPγ plasmid, C/EBPδ plasmid, and MAF plasmid encode mouse C/EBPα, C/EBPβ, C/EBPγ, C/EBPδ, and MAF, respectively, under control of the CMV promoter; they were generated by subcloning the coding regions of C/EBPα (NM_007678.3), C/EBPβ (NM_009883.4), C/EBPγ (NM_009884.3), C/EBPδ (NM_007679.4), and MAF (NM_001025577.2) into pcDNA3.1. The LUZP1–FLAG plasmid and the BRD4–FLAG plasmid encode mouse LUZP1 and the long isoform of mouse BRD4, respectively, each with three copies of the FLAG epitope tag at the N terminus under control of the CMV promoter; they were generated by subcloning the coding regions of LUZP1 (NM_024452.2) and BRD4 (NM_001286630.1) into the p3X-FLAG-CMV-10 plasmid (Sigma). The WT *Gadd45a* exon 4 reporter plasmid was generated by amplifying a fragment of the mouse *Gadd45a* gene (+1733 to +1933 bp relative to the transcription start site) using mouse skeletal muscle genomic DNA and the following primers: 5′-ATCTCCCGGAACGGTGAT-3′ (sense) and 5′-TCTTCAGGCTCACCTCTC-3′ (antisense); the amplified fragment was then cloned into the KpnI and XhoI sites in the pGL3-Basic firefly luciferase reporter vector (Promega). The mutant *Gadd45a* exon 4 reporter plasmid was generated by site-directed mutagenesis of the ATF4–C/EBP composite site in the *Gadd45a* exon 4 reporter plasmid, changing the ATF–C/EBP site from TGATGCAA to CAGAATGG. The pRL-CMV *Renilla* luciferase control plasmid was from Promega. Recombinant adenoviruses expressing GFP plus FLAG-tagged ATF4 or GFP plus FLAG-tagged ATF4ΔbZIP were described previously (2).

### Mouse protocols

The mice used in these studies were males from the C57BL/6 strain, obtained from Charles River at ages 6–8 weeks old and used for experiments within 2 weeks of their arrival. Mice were housed (up to five mice per cage) in ventilated cages (Thoren Rack system, no. 9 size cages) at 21 °C with 12:12-h light/dark cycles and *ad libitum* access to standard chow (Harlan-Teklad formula 7913) and water (filtered automatic watering system). Transfection of mouse skeletal muscle with plasmid DNA was performed as described previously (5). Briefly, mice were anesthetized with 91 mg/kg ketamine and 9.1 mg/kg xylazine; hind limbs were shaved; and the tibialis anterior muscles (TAs) were injected with 30 μl of 0.4 units/μl bovine placental hyaluronidase (Sigma) resuspended in sterile 0.9% saline. Two hours later, mice were re-anesthetized. The TAs were then injected with plasmid DNA in sterile saline, coated with ultrasound jelly, and subjected to 10 20-ms pulses of 175 V/cm using an ECM-830 electroporator (BTX Harvard Apparatus). Following transfection, mice were returned to their cages to resume normal activities for 3–10 days before muscle harvest, as noted in the figure legends. Unilateral TA muscle immobilization was performed as described previously (2). Euthanasia was performed by subjecting animals to CO_2_ exposure (flow rate of 3 liters/min) until breathing stopped for a period of 1 min, and euthanasia was confirmed by decapitation. Euthanasia methods were approved by the Panel on Euthanasia of the American Veterinary Medical Association. All animal procedures were approved by the Institutional Animal Care and Use Committee of the University of Iowa.

### TAP

Freshly-excised mouse TA muscles were immediately frozen in liquid N_2_ and stored at −80 °C. To prepare protein lysates, groups of four TA muscles transfected with either empty TAP plasmid or ATF4 bZIP TAP plasmid were placed in 17 × 100-mm Falcon tubes containing 2 ml of ice-cold Buffer A, and then homogenized with an OMNI TH-01 tissue tearer. Homogenates were then moved to 2-ml microcentrifuge tubes, rotated for 2 h at 4 °C, and then centrifuged at 12,000 × *g* for 30 min. The soluble fractions from each group (control and ATF4 bZIP) were then pooled in one 15-ml conical tube per group. The protein concentration of each sample was then obtained using the BCA method, followed by addition of 3 μl of pre-washed anti-FLAG magnetic beads per mg of protein. We then rotated the samples for 2 h at 4 °C, captured the magnetic beads with a magnetic separator, removed and discarded the supernatant, and briefly washed the beads four times on ice with 6 ml of ice-cold Buffer B. To elute bound proteins, we incubated and rotated the beads in 3 ml of Buffer B containing 150 μg/ml 3X-FLAG peptide for 30 min at 4 °C, captured the beads with a magnetic separator, moved the supernatant to a fresh 15-ml tube on ice, briefly incubated the beads two more times with 3 ml of ice-cold Buffer B, and combined these two additional supernatants with the first supernatant containing 3X-FLAG peptide. We then added 2 μl of pre-washed S-protein–agarose per mg of protein, incubated the samples on a belly shaker for 1 h at 4 °C, centrifuged the samples at 200 × *g* for 1 min, briefly washed the pellets three times on ice with 1.2 ml of ice-cold Buffer B, resuspended the pellets in 1.2 ml of ice-cold Buffer B, transferred the samples to 1.5 ml of LoBind microcentrifuge tubes (Eppendorf), and centrifuged at 200 × *g* for 1 min. We then removed and discarded the supernatants, resuspended the pellets in 0.4 ml of ice-cold Buffer C, incubated the samples for 5 min at 65 °C, centrifuged the samples at 200 × *g* for 1 min, and then transferred the supernatants to fresh 1.5-ml LoBind microcentrifuge tubes on ice. We then repeated this process two more times (resuspending the beads in 0.4 ml of ice-cold Buffer C, incubating them for 5 min at 65 °C, and centrifuging them at 200 × *g* for 1 min), each time transferring the resulting supernatant to the first supernatant in the 1.5-ml microcentrifuge tube on ice. The two pulldown samples (each containing the combined final supernatants from either control or ATF4 muscles) were then centrifuged at 13,000 × *g* for 1 min at 4 °C to remove any residual beads, and the resulting supernatants were then concentrated to 75 μl with Ultra Ultracel-3K columns (Amicon). We then added 25 μl of NuPAGE 4× LDS sample buffer to each sample and stored the samples at −80 °C. An aliquot of each pulldown sample (10 μl) was subsequently incubated for 5 min at 95 °C, subjected to SDS-PAGE on 4–12% NuPAGE gels (Invitrogen), and then visualized with SilverQuest Silver Staining kit (Invitrogen). The remainder of each pulldown sample (90 μl) was used for mass spectrometric studies, as described below.

### Preparation of TAP enrichment samples for mass spectrometry

To remove detergents and other noncompatible reagents, protein samples were subjected to a “stack” 1D SDS-polyacrylamide gel clean-up procedure as follows: after TAP pulldown and elution, samples were added to 10 μl of NuPAGE LDS Sample Buffer (four times) and incubated at 70 °C for 10 min. Samples were then loaded into a NuPAGE 4–12% BisTris gel, run only until the dye front had progressed about 1 cm into the gel, and visualized with GelCode Blue Stain Reagent (Life Technologies, Inc.). Gel slices were then excised from the 1-cm–long, protein-containing gel range, destained, reduced with a final concentration of 10 mm DTT at 56 °C, and alkylated with 55 mm iodoacetamide at room temperature in the dark. In-gel trypsin digestion was performed using a 1:20 enzyme to protein ratio overnight at 37 °C. Resulting peptides were extracted and desalted using C-18 zip-tips (Millipore, Billerica, MA).

### Proteolytic digestion of TAP enrichment samples

For mass spectrometric analysis, proteins were reduced with 20 mm DTT (37 °C for 1 h) and subsequently alkylated with 40 mm iodoacetamide (30 min at room temperature in the dark). Samples were diluted 10-fold with 100 mm Tris, pH 8.0, and incubated overnight at 37 °C with sequencing grade trypsin (Promega) added at a 1:50 enzyme/substrate ratio (w/w). Prior to mass spectrometric analysis, the acetylated peptide enrichment samples were concentrated and desalted using C-18 zip-tips (Millipore, Billerica, MA).

### Mass spectrometric analysis

Samples were analyzed by reverse-phase HPLC–ESI-MS/MS using an Eksigent Ultra Plus nano-LC 2D HPLC system (Dublin, CA) with a cHiPLC system (Eksigent) that was directly connected to a quadrupole TOF TripleTOF 6600 mass spectrometer (SCIEX, Concord, Ontario, Canada) (57, 58). After injection, peptide mixtures were loaded onto a C18 pre-column chip (200-μm × 0.4-mm ChromXP C18-CL chip, 3 μm, 120 Å, SCIEX) and washed at 2 μl/min for 10 min with the loading solvent (H_2_O, 0.1% formic acid) for desalting. Subsequently, peptides were transferred to the 75-μm × 15-cm ChromXP C18-CL chip, 3 μm, 120 Å (SCIEX), and eluted at a flow rate of 300 nl/min with a 2-h gradient using aqueous and acetonitrile solvent buffers (57, 59). For peptide and protein identifications, the mass spectrometer was operated in data-dependent acquisition (DDA) mode, where the 30 most abundant precursor ions from the survey MS1 scan (250 ms) were isolated at 1 *m/z* resolution for collision-induced dissociation tandem MS (CID-MS/MS, 100 ms per MS/MS, “high sensitivity” product ion scan mode) using the Analyst 1.7 (build 96) software with a total cycle time of 3.3 s, as described previously (59, 60). For quantification, all peptide samples were analyzed by data-independent acquisition (DIA, *e.g.* SWATH) (61), using 64 variable-width isolation windows (62). The variable window width is adjusted according to the complexity of the typical MS1 ion current observed within a certain *m*/*z* range using a DIA “variable window method” algorithm (more narrow windows were chosen in “busy” *m*/*z* ranges and wide windows in *m*/*z* ranges with few eluting precursor ions). DIA acquisitions produce complex MS/MS spectra, which are a composite of all the analytes within each selected Q1 *m*/*z* window. The DIA cycle time of 3.2 s included a 250-ms precursor ion scan followed by 45 ms accumulation time for each of the 64 variable SWATH segments.

### Mass spectrometric data processing and bioinformatics

Mass spectrometric DDAs were analyzed using the database search engine ProteinPilot (63) (SCIEX Beta 4.5, revision 1656) using the Paragon algorithm (4.5.0.0,1654). The following sample parameters were used: trypsin digestion, cysteine alkylation set to iodoacetamide, urea denaturation, and species *Mus musculus*. All data files were searched using the SwissProt Sprot_2016_06.fasta database with a total of 16,794 *M. musculus* protein sequences. A cutoff peptide confidence value of 99 was chosen. The Protein Pilot false discovery rate (FDR) analysis tool algorithm (63) provided a global FDR of 1% and a local FDR at 1% in all cases. We then subjected the MS data to an informatics workflow to identify high-confidence ATF4 interactors, which were defined as proteins that: 1) were present in the ATF4 bZIP pulldown sample in at least one of the two independent pulldown experiments described here; 2) were not present in control pulldown samples (empty TAP construct) in the two independent pulldown experiments described here or in nine other independent pulldown experiments; and 3) did not interact with five other bait proteins that we have studied by similar methods (Gadd45a (14) and four others not yet published). For database searches, a cutoff peptide confidence value of 99 was chosen, and typically, a minimum of two identified peptides per protein was required. For the assessment of “One-Peptide Wonders” (few proteins for which only one unique peptide was identified), the corresponding peptide MS/MS spectra were inspected for quality, and the corresponding spectral library was uploaded to Panorama as described below. Fig. S2 shows selected “one-peptide wonders” and the MS/MS from key interacting proteins that were inspected for quality; the corresponding spectral library was uploaded to Panorama as described below. Briefly, the MS/MS spectra were inspected “manually” based on an adaptation of previously published criteria (64). Proteins with only one observed peptide were validated and added to the list only if the following criteria were satisfied. 1) The peptide represented a unique peptide in the human proteome as confirmed by BLAST searches. 2) Manual inspection of the spectrum verified the following criteria: (*a*) good signal-to-noise (signal-to-noise >3, for the majority of fragment ions); (*b*) a minimum of three consecutive y- or b-ions; and (*c*) any y-ion with an N-terminal proline must be intense. In addition, we also compared MS/MS spectra with spectral libraries at the Global Proteome Machine.

### Raw data accession and panorama spectral libraries

The raw and processed data associated with this study can be downloaded from MassiVE (UCSD) (MassIVE number MSV000084538 and ProteomeXChange number PXD016139). Details for peptide and protein identifications are provided as excel files as well as summarized in Table S1 and also see Fig. S2 with selected tandem mass spectra of key ATF4-interacting proteins. A spectral library with MS/MS spectra for all peptides/proteins was transferred and published to the interactive, web-based data sharing Panorama server (64, 65). The spectral viewer may be accessed at https://panoramaweb.org/targetedms/Schilling/Chris_Adams_ATF4_interactome/showList.view.^3^

### Immunoblot analyses

Skeletal muscles were snap-frozen in liquid nitrogen and homogenized in ice-cold Buffer D using a Precellys 24 (Bertin Technologies) homogenizer at 6000 rpm for 30 s per cycle for three cycles. The muscle homogenate was rotated for 1 h at 4 °C and centrifuged at 16,000 × *g* for 20 min at 4 °C, and then the supernatant was removed for BCA analyses and SDS-PAGE, as described below. Myotube protein extracts were prepared by scraping PBS-washed myotubes into cold lysis Buffer D supplemented with complete mini-EDTA–free protease inhibitor (Roche Applied Science) and PhosStop phosphatase inhibitor (Roche Applied Science) according to the manufacturer's instructions. An aliquot of each protein sample was used to determine protein concentration by the BCA method (Pierce), and another aliquot was mixed with 0.25 volume of Buffer E and heated at 95 °C for 5 min. An equal amount of protein from each sample was subjected to SDS-PAGE and then transferred to 0.45-μm nitrocellulose membranes (Bio-Rad). Immunoblots were performed at 4 °C for 16 h. HRP-conjugated anti-FLAG IgG was diluted to 1:1000 in Fig. 3, 1:5000 in Fig. 5, and 1:20,000 in Figs. 2 and 7. Antibodies against C/EBPα, C/EBPβ, C/EBPγ, C/EBPδ, and MAF were diluted to 1:1000 and were followed by HRP-conjugated anti-mouse IgG or HRP-conjugated anti-rabbit IgG. The anti-LUZP1 antibody was diluted to 1:2000 and was followed by HRP-conjugated anti-rabbit IgG. Bound antibodies were visualized by chemiluminescence (SuperSignal West Pico; Thermo Fisher Scientific).

### Histological analyses of mouse skeletal muscle

Harvested muscles were immediately fixed in 4% (w/v) paraformaldehyde for 16 h at 4 °C, and then incubated in 30% sucrose (w/v) for 24 h. The muscles were then embedded in Tissue Freezing Medium (Triangle Biomedical Sciences), and a Microm HM 505E cryostat was used to prepare 10-μm sections from the muscle midbelly. Sections were washed with PBS three times and then mounted with Vectashield (Vector Laboratories). All sections were examined and photographed using a Nikon Eclipse Ti automated inverted microscope equipped with NIS-Elements BR digital imaging software. Image analysis was performed using ImageJ software. Skeletal muscle fiber size was analyzed by measuring the lesser diameter (minimal *Feret* diameter) of muscle fibers, as recommended elsewhere (65).

### Quantitative real-time RT-qPCR

Mouse skeletal muscle RNA was extracted using TRIzol solution (Invitrogen) and purified with the Turbo DNA-free kit (Ambion) (5). Myotube RNA was extracted using TRIzol and purified using the RNeasy kit and RNase-free DNase Set (Qiagen) (2). qPCR of skeletal muscle and myotube RNA was performed as described previously (2, 5) using a high-capacity cDNA reverse transcription kit (Applied Biosystems). qPCR studies were performed with a Quant Studio 6 Flex Real-time PCR system (Applied Biosystems) using TaqMan Gene Expression Assays (Applied Biosystems). All qPCR samples were run in triplicate, and the cycle threshold (*C_t_*) values were averaged. For data analysis, the ΔΔ*C_t_* method was utilized, with *36B4* mRNA serving as the invariant control.

### Reporter gene assay

Mouse skeletal muscles were transfected with reporter gene plasmids and harvested as described in Fig. 7 legend. Harvested muscles were placed in 1 ml of 1× Passive Lysis Buffer (Promega) and then homogenized with three 30-s cycles of a Precellys 24 (Bertin Technologies) homogenizer set at 6000 rpm. The homogenate was rotated for 20 min at 4 °C and centrifuged at 2000 × *g* for 5 min at 4 °C, and then 20 μl of the sample supernatant was mixed with 100 μl of Luciferase Assay Reagent II (Promega), followed by quantification of firefly luciferase activity with a Synergy H1 microplate reader (BioTek). Firefly luciferase activity was then quenched, and *Renilla* luciferase activity was activated by adding 100 μl of Stop & Glo Reagent (Promega). Mean firefly luciferase activity was then normalized to mean *Renilla* luciferase activity to give the final results.

### Cell culture

Mouse C2C12 myoblasts were obtained from ATCC (CRL-1772) and maintained at 37 °C and 5% CO_2_ in Dulbecco's modified Eagle's medium (DMEM) (ATCC catalogue no. 30-2002) containing antibiotics (100 units/ml penicillin, 100 μg/ml streptomycin sulfate) and 10% (v/v) fetal bovine serum (FBS). Myoblasts were set up for experiments on day 0 in 6-well plates at a density of 2.5 × 10^5^ cells/well. On day 2, differentiation was induced by replacing 10% FBS with 2% horse serum. On day 7, cells were rinsed once with PBS, and then 1 ml of DMEM containing adenovirus (multiplicity of infection 250) was added to each well. Two hours later, 1 ml of DMEM containing 1% horse serum plus antibiotics was added to each well. Infection efficiency was >90%. Cells were used for experiments between 6 and 48 h post-adenovirus treatment, as indicated in Fig. 7 legend.

### Chromatin immunoprecipitation (ChIP)

Following a 24-h infection with recombinant adenovirus expressing either GFP plus ATF4ΔbZIP or GFP plus ATF4, C2C12 myotubes were treated with 10 μm calpain inhibitor I (ALLN, Sigma catalogue no. A6185) for 15 min and then fixed with 1% formaldehyde suspended in DMEM for 10 min at room temperature with gentle rocking. Excess formaldehyde was quenched with 125 μm glycine for 5 min with gentle rocking at room temperature. Myotubes were then washed two times with ice-cold PBS and collected. DNA was sonicated with a Qsonica Sonicator (Model Q55) and conditions that were empirically determined to cleave genomic DNA into 250–800-bp fragments (seven 10-s pulses at an amplitude of 40, performed 30 s apart with samples in an ice-water bath throughout). ChIP was performed using mouse polyclonal anti-IgG (Millipore, catalogue no. 12-371B), mouse monoclonal anti-FLAG IgG (Sigma, catalogue no. F1804), mouse monoclonal anti-C/EBPβ IgG (Biolegend, catalogue no. 606202), and the EZ-ChIP kit (Millipore 17-371R) according to the manufacturer's instructions. ChIP-qPCR was carried out using a 7500 Fast Real-Time PCR System (Applied Biosystems). All qPCRs were performed in triplicate, and the *C_t_* values were averaged to give the final results. All reactions were performed in 20-μl reaction volumes using POWER SYBR Green Master (Thermo Fisher Scientific, catalogue no. 4367659) with 10% betaine and sense and antisense primers at 400 nm. Primers were designed to amplify the portion of the *Gadd45a* gene that was previously analyzed by the reporter assay experiments. Primer sequences were 5′-ATCTCCCGGAACGGTGAT-3′ (sense) and 5′-TCTTCAGGCTCACCTCTC-3′ (antisense). Cycling conditions included an initial denaturation phase of 50 °C for 2 min and 95 °C for 10 min, followed by the amplification phase of 40 cycles of 95 °C for 15 s and 59 °C for 1 min; and a final denaturation phase was completed for melt curve analysis. Quantification was completed by fold enrichment where ChIP signal was calculated as a fold increase in signal relative to the background IgG signal.

### Statistics

Statistical analyses were performed with GraphPad Prism. The statistical tests and sample sizes are provided in the figure legends.

## Author contributions

S. M. E., B. S., and C. M. A. conceptualization; S. M. E., S. A. B., N. B., G. R. M., Z. P. S., J. M. D., A. A.-Z., K. C. T., A. D. D., J. A. R., S. C. B., B. S., and C. M. A. formal analysis; S. M. E., S. A. B., N. B., G. R. M., Z. P. S., J. M. D., A. A.-Z., K. C. T., A. D. D., J. A. R., S. C. B., B. S., and C. M. A. investigation; S. M. E., B. S., and C. M. A. writing-original draft; S. M. E., S. A. B., N. B., G. R. M., Z. P. S., J. M. D., A. A.-Z., K. C. T., A. D. D., J. A. R., S. C. B., B. S., and C. M. A. writing-review and editing; S. M. E., B. S., and C. M. A. funding acquisition; S. M. E., B. S., and C. M. A. project administration.

## Supplementary Material

Supporting Information

## Footnotes

3 Please note that the JBC is not responsible for the long-term archiving and maintenance of this site or any other third party hosted site.

## Footnotes

2 The abbreviations used are:

ATF4: activating transcription factor 4
HRP: horseradish peroxidase
qPCR: quantitative PCR
ANOVA: analysis of variance
TA: tibialis anterior
C/EBPβ: CCAAT enhancer-binding protein β
eGFP: enhanced GFP
CMV: cytomegalovirus
BisTris: 2-[bis(2-hydroxyethyl)amino]-2-(hydroxymethyl)propane-1,3-diol
DMEM: Dulbecco's modified Eagle's medium
FBS: fetal bovine serum
DIA: data-independent acquisition
FDR: false discovery rate
bZIP: basic leucine zipper
TAP: tandem affinity purification
LIP: liver inhibitory protein
LAP: liver-activating protein
EmGFP: emerald GFP.
