# Supplementary material for: Activating transcription factor 4 (ATF4) promotes skeletal muscle atrophy by forming a heterodimer with the transcriptional regulator C/EBPβ
Source: J Biol Chem. 2020 Jan 17;295(9):2787–803. doi: 10.1074/jbc.RA119.012095 (PMC7049960; doi:10.1074/jbc.RA119.012095)
Supplement: Supporting Information [file supp_295_9_2787__index.html]

Activating transcription factor 4 (ATF4) promotes skeletal muscle atrophy by forming a heterodimer with the transcriptional regulator C/EBPβ — ATF4–C/EBPβ heterodimer promotes skeletal muscle atrophy — Supporting Information 

# Activating transcription factor 4 (ATF4) promotes skeletal muscle atrophy by forming a heterodimer with the transcriptional regulator C/EBPβ

## Supporting Information

- Supporting Information PDF - Mass spectrometry data
- Supplemental Table1
