# Supplementary material for: Activating transcription factor 4 (ATF4) promotes skeletal muscle atrophy by forming a heterodimer with the transcriptional regulator C/EBPβ
Source: J Biol Chem. 2020 Jan 17;295(9):2787–803. doi: 10.1074/jbc.RA119.012095 (PMC7049960; doi:10.1074/jbc.RA119.012095)
Supplement: Supporting Information [file supp_RA119.012095_157402_1_supp_459761_q47wy6.pdf]

SUPPORTING INFORMATION

Supplemental Table 1: Proteomics data.

Supplemental Figure 1: In vivo transfection of mouse skeletal muscle. Mouse tibialis anterior (TA) muscles were transfected in vivo with 5 µg plasmid encoding eGFP, then harvested at the indicated times post-transfection. Images are of the entire TA muscles, which were photographed at low magnification to visualize eGFP expression.

Supplemental Figure 2. Selected MS/MS spectra from key ATF4-interacting proteins. (A) MS/MS for peptide from CEBPB CCAAT/enhancer-binding protein beta (P28033): peptide VLELTAENER with the precursor ion at m/z 587.31 $^{2+}$. (B) MS/MS for peptide from CEBPB CCAAT/enhancer-binding protein beta (P28033): peptide APAAEPAIGEHER with the precursor ion at m/z 674.34 $^{2+}$. (C) MS/MS for peptide from CEBPD CCAAT/enhancer-binding protein delta (Q00322): peptide GSPGPSLAPGTVR with the precursor ion at m/z 598.33 $^{2+}$. (D) MS/MS for peptide from CEBPD CCAAT/enhancer-binding protein delta (Q00322): peptide LVELSAENEK with the precursor ion at m/z 566.30 $^{2+}$. (E) MS/MS for peptide from CEBPA CCAAT/enhancer-binding protein alpha (P53566): peptide GLAGAHPDLR with the precursor ion at m/z 503.78 $^{2+}$. (F) MS/MS for peptide from CEBPG CCAAT/enhancer-binding protein gamma (P53568): peptide *Q*KAQDTLQR with the precursor ion at m/z 535.78 $^{2+}$. (G) MS/MS for peptide from MAF MOUSE Transcription factor Maf (P54843): peptide FSDEQLVTMSVR with the precursor ion at m/z 714.35 $^{2+}$. (H) MS/MS for peptide from LUZP1 Leucine zipper protein 1 (Q8R4U7): peptide ISSELEMLR with the precursor ion at m/z 539.28 $^{2+}$. (I) MS/MS for peptide from BRD4 Bromodomain-containing protein 4 (Q9ESU6): peptide A*Q*AEHAEKEK with the precursor ion at m/z 571.28 $^{2+}$. (J) MS/MS for peptide from PTRF Polymerase I and transcript release factor, Cavin-1 (O54724): peptide ATEEPSGTGSDELIK with the precursor ion at m/z 767.37 $^{2+}$. (K) MS/MS for peptide from SDPR Serum deprivation-response protein, Cavin-2 (Q63918): peptide YQASTSNTVSK with the precursor ion at m/z 593.29 $^{2+}$. (L) MS/MS for peptide from MURC Muscle-related coiled-coil protein, Cavin-4 (A2AMM0): peptide VASVVDSVQASQKR with the precursor ion at m/z 491.94 $^{3+}$. (M) MS/MS for peptide from SYNCI Syncoilin (Q9EPM5): peptide ALQDTQAEIQE*M*R with the precursor ion at m/z 774.87 $^{3+}$.

Supplemental Figure 3. Mutation of the ATF-C/EBP site abolishes immobilization-induced Gadd45a reporter activity. Bilateral mouse TA muscles were transfected with 5 µg mutant reporter plasmid (described in Figure 7) plus 0.3 µg Renilla plasmid. Three days after transfection, one hindlimb in each mouse was immobilized. Six days post-transfection, bilateral TAs were harvested for analysis of reporter gene activity. In each muscle, the level of reporter gene activity was normalized to the average level in the transfected mobile TAs. Each circle represents one transfected muscle, and bars denote the means. *P*-value was determined with a t-test.

Figure S1

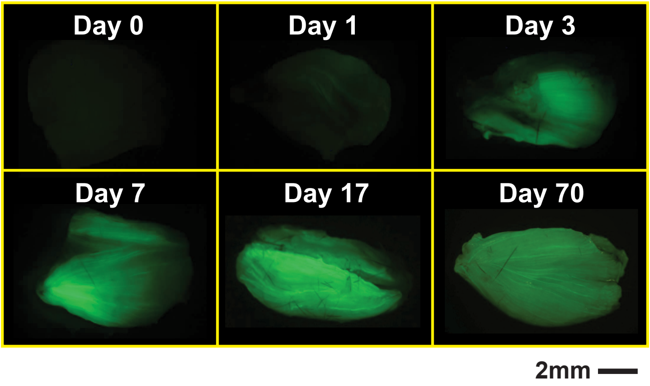

Suppl Fig 2A

CEBPB CCAAT/enhancer-binding protein beta

P28033 VLELTAENER [253, 262] m/z 587.31 2+

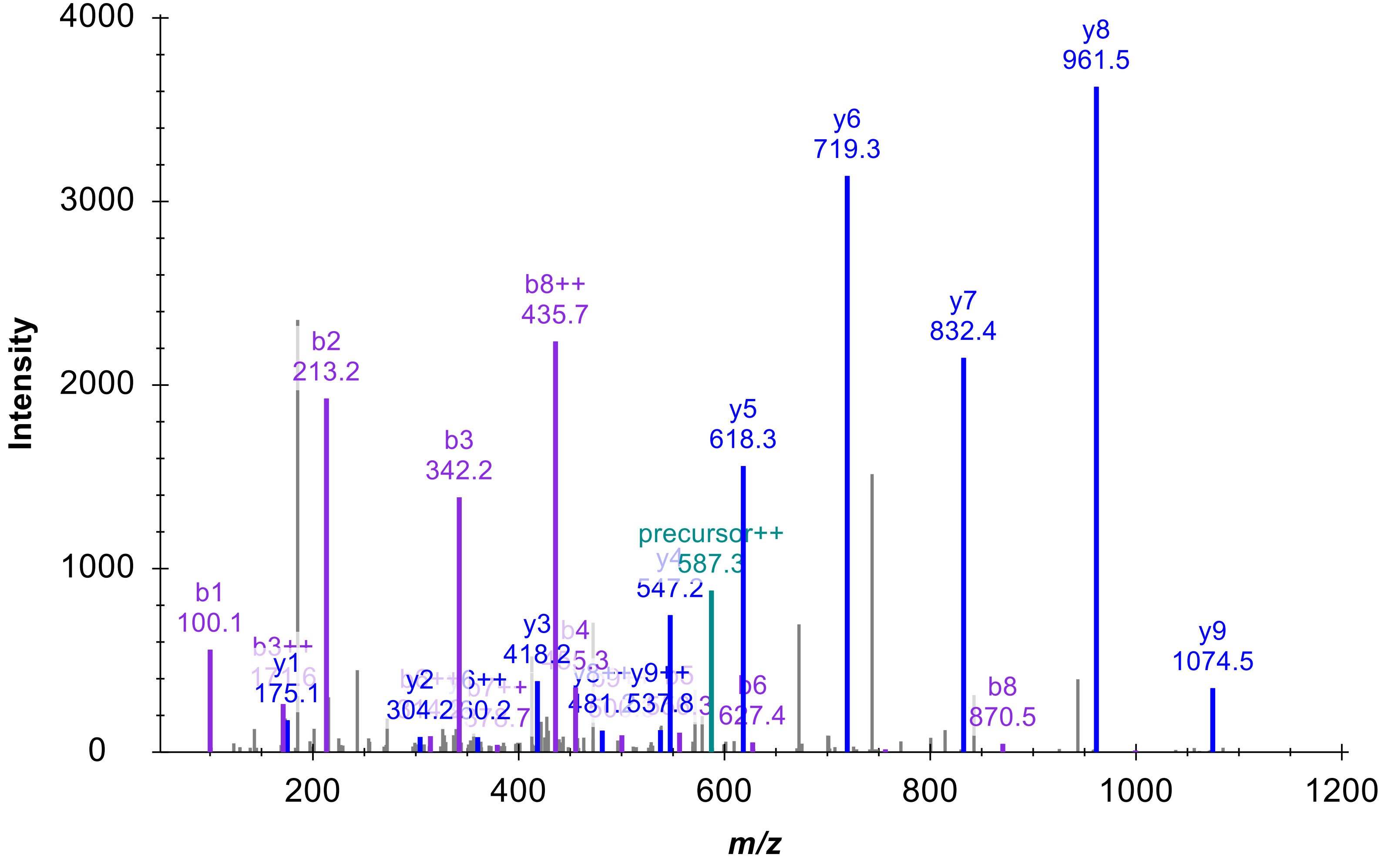

Suppl Fig 2B

CEBPB CCAAT/enhancer-binding protein beta

P28033 APAAAEPAIGEHER (46 - 58) m/z 674.34 $^{2+}$

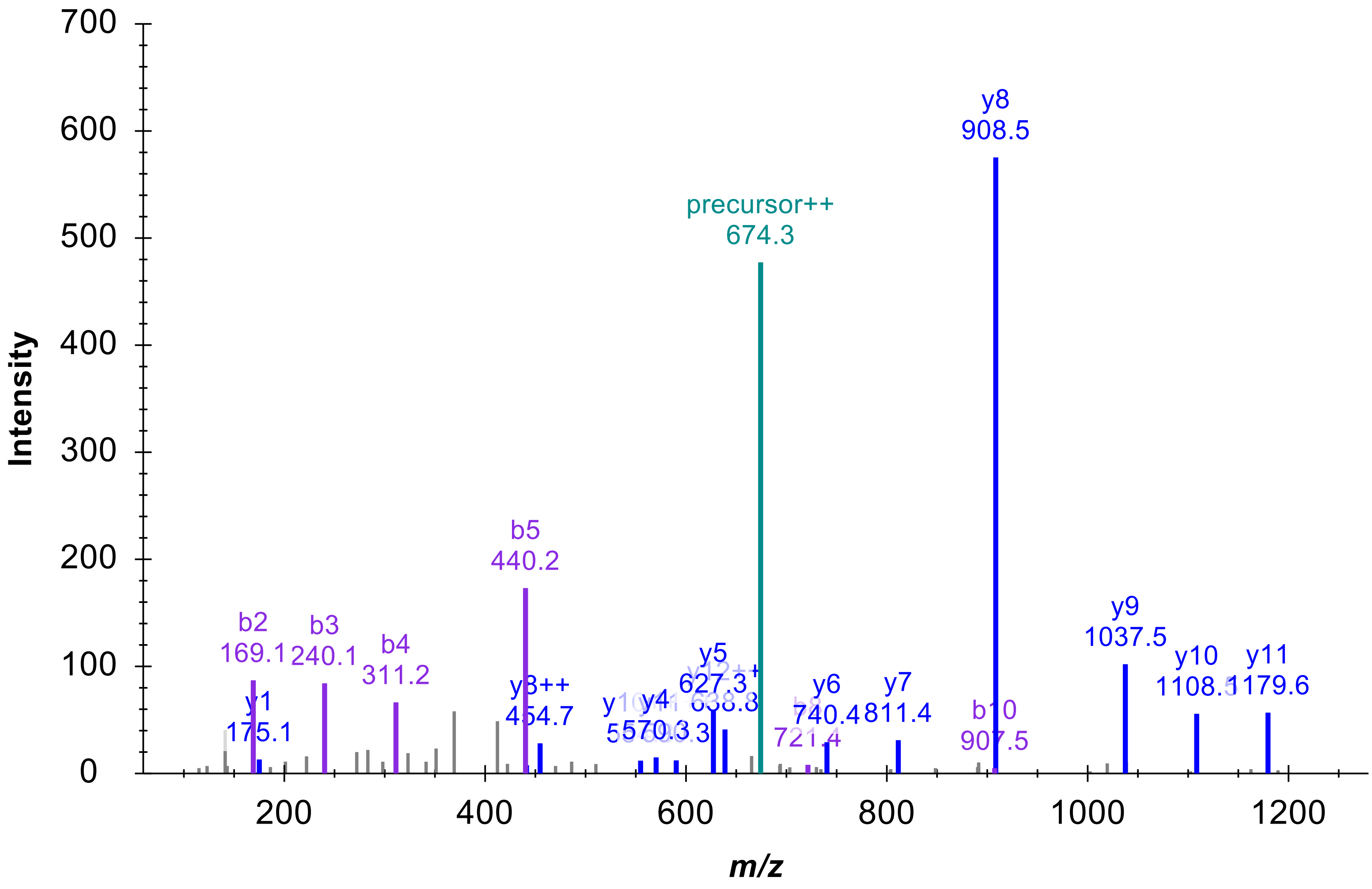

Suppl Fig 2C

CEBPD CCAAT/enhancer-binding protein delta

Q00322 GSPGPSLAPGTVR [165, 177] m/z 598.33 $^{2+}$

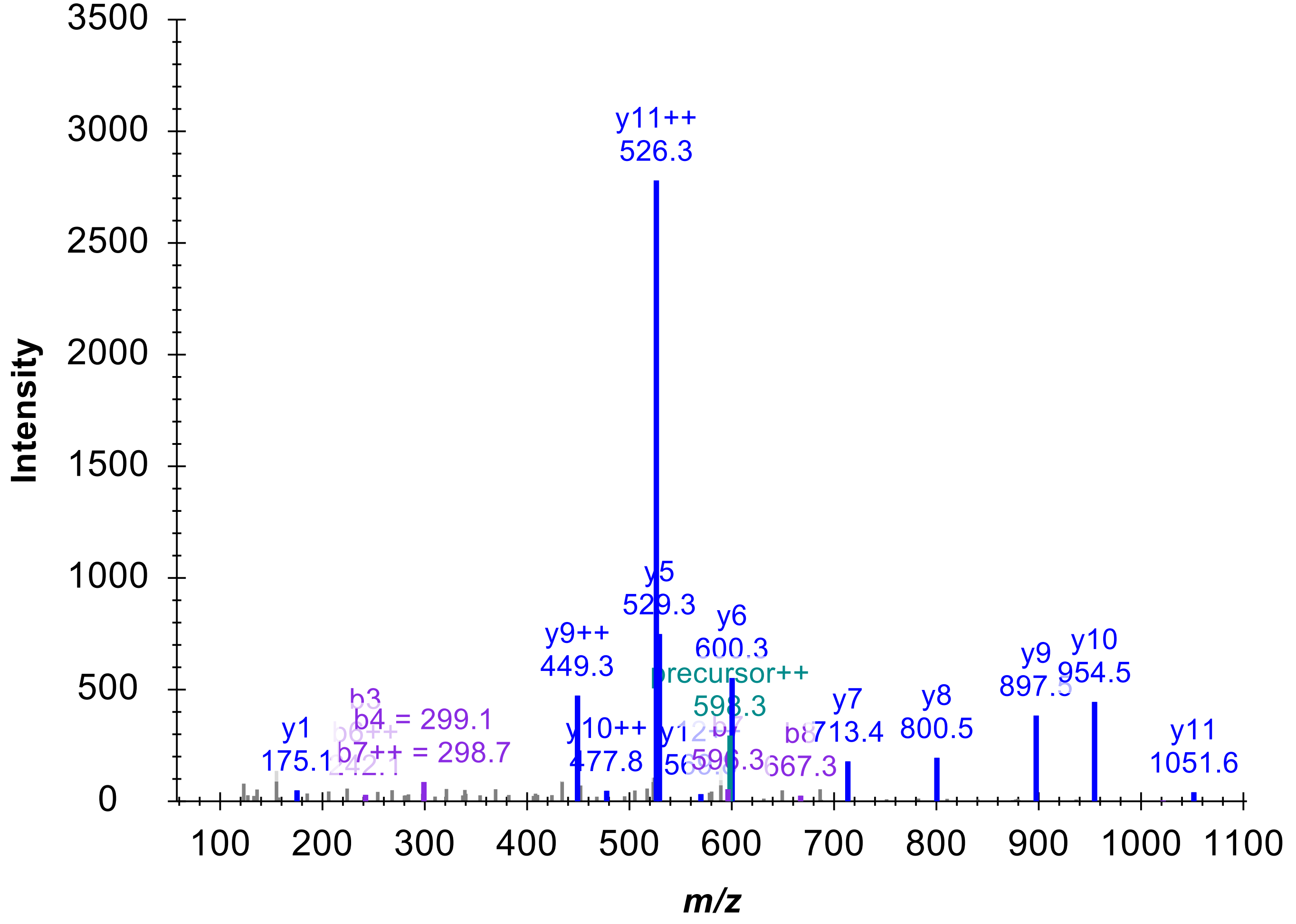

Suppl Fig 2D

CEBPD CCAAT/enhancer-binding protein delta

Q00322 LVELSAENEK (223-232) m/z 566.30 $^{2+}$

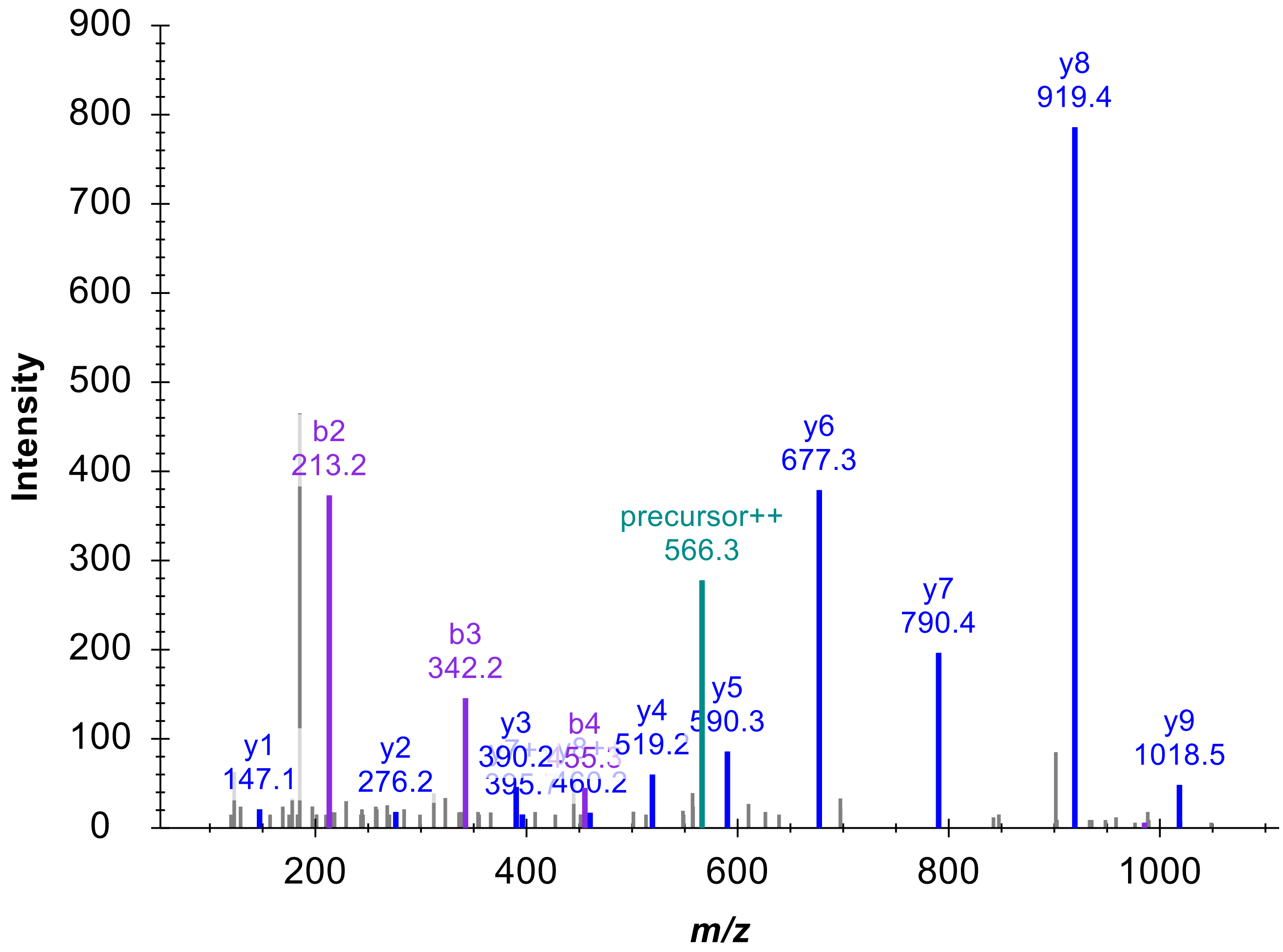

Suppl Fig 2E

CEBPA CCAAT/enhancer-binding protein alpha

P53566 GLAGAHPDLR (251, 260) m/z 503.78 $^{2+}$

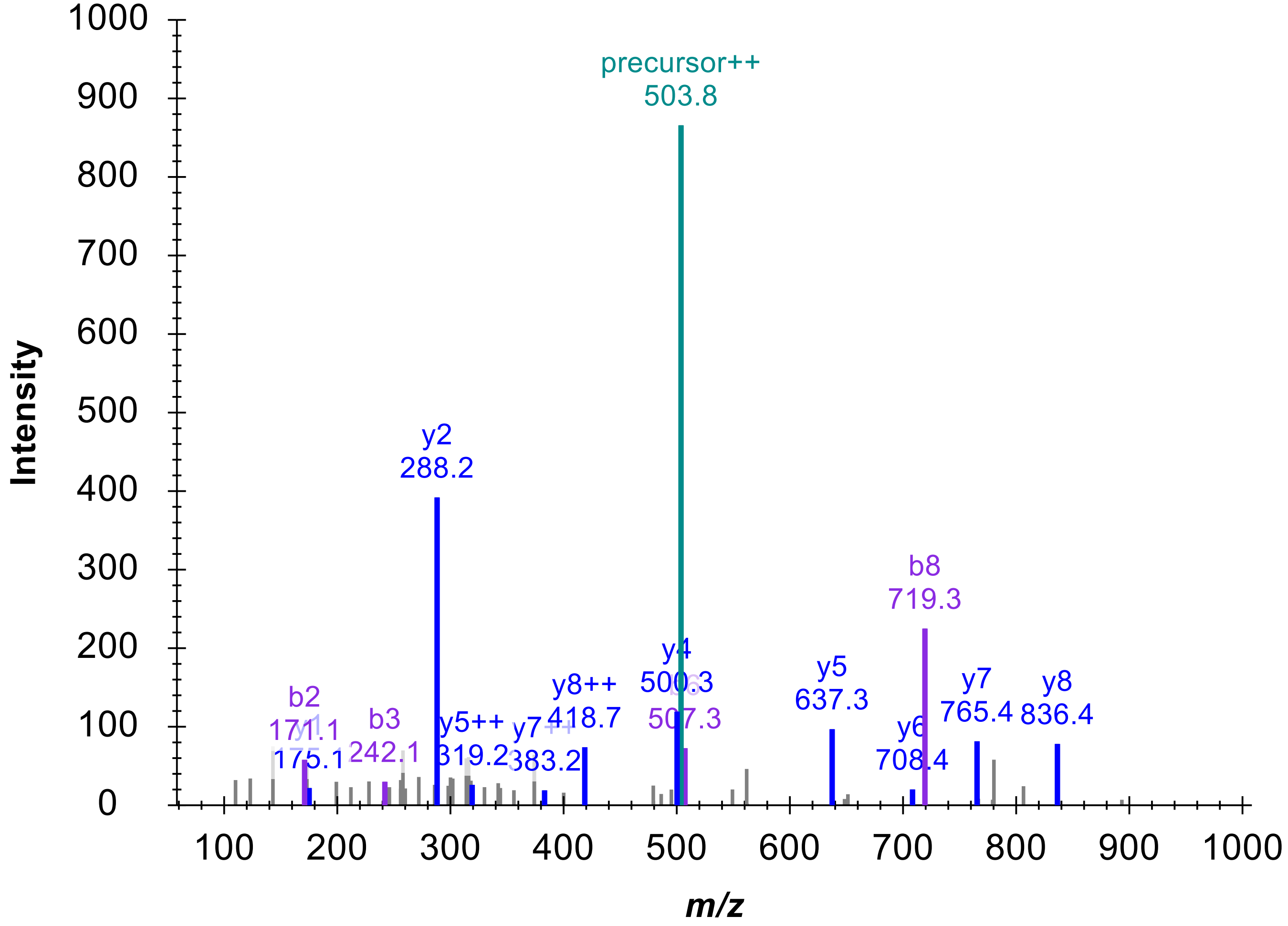

Suppl Fig 2F

CEBPG CCAAT/enhancer-binding protein gamma

P53568 *Q*KAQDTLQR (85, 93) m/z 535.78 $^{2+}$

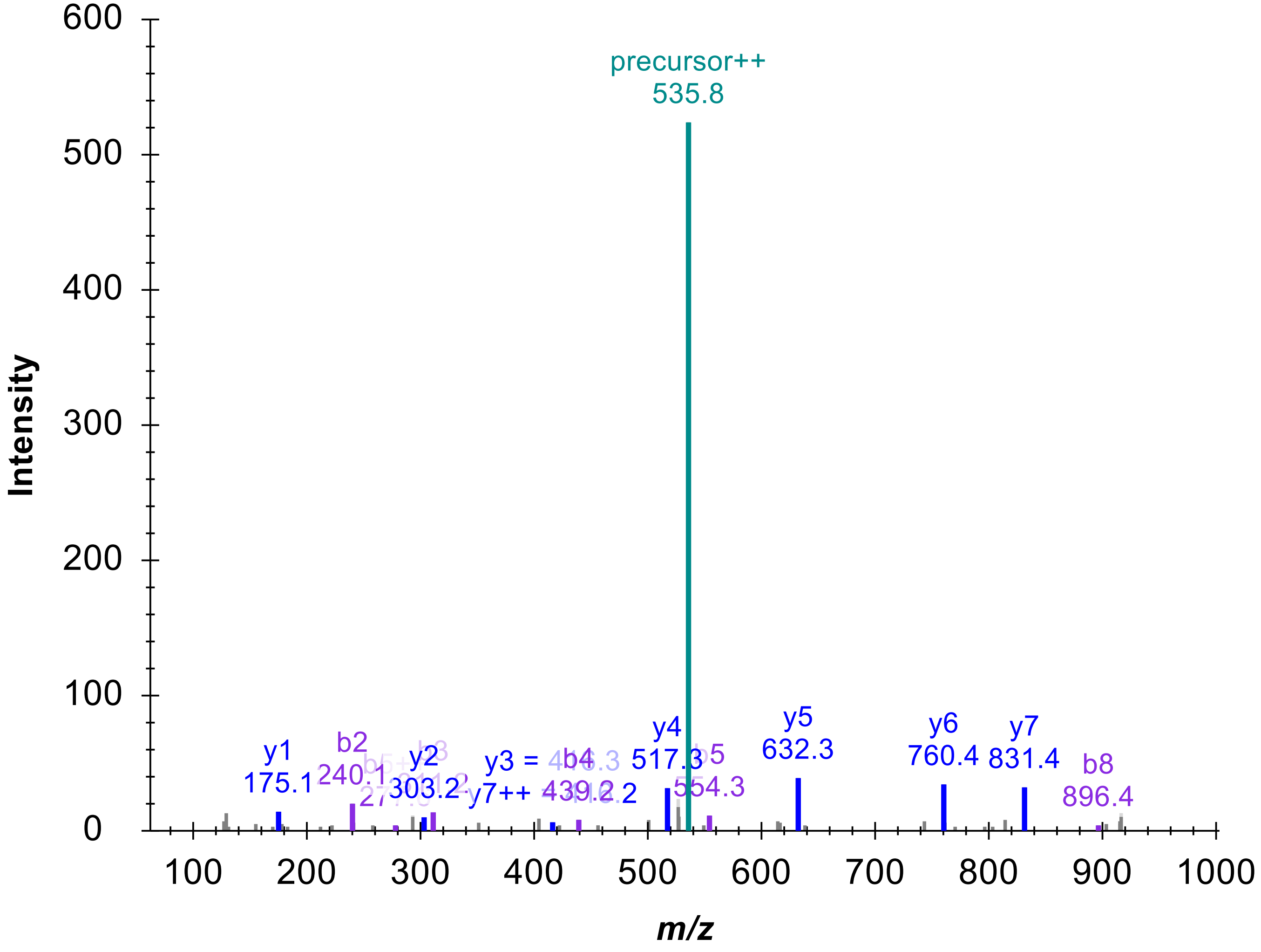

Suppl Fig 2G

MAF Transcription factor Maf

P54843 FSDEQLVTMSVR [257, 268] m/z 714.35 $^{2+}$

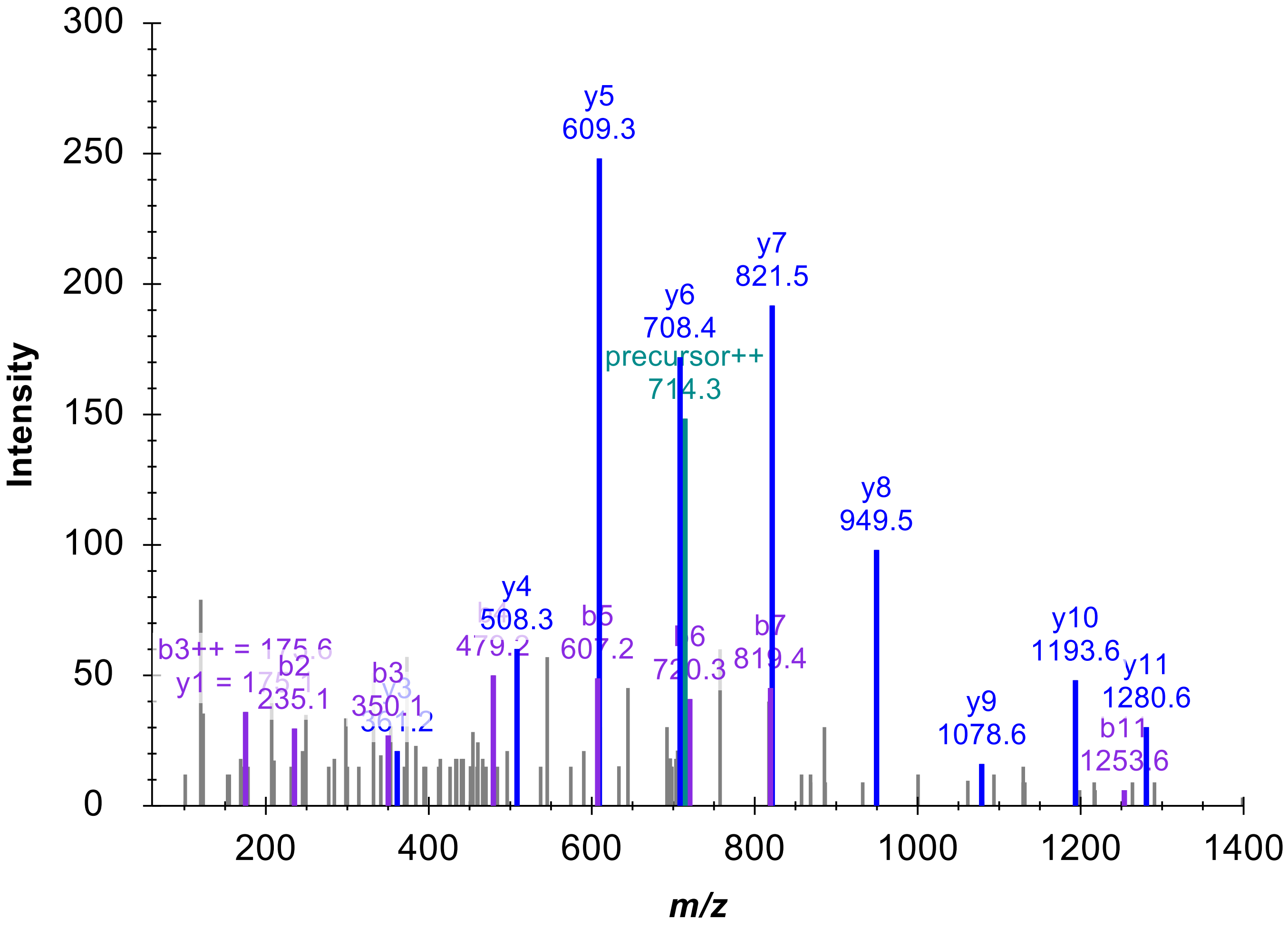

Suppl Fig 2H

LUZP1 Leucine zipper protein 1

Q8R4U7 ISSELEMLR [150, 158] m/z 539.28 2+

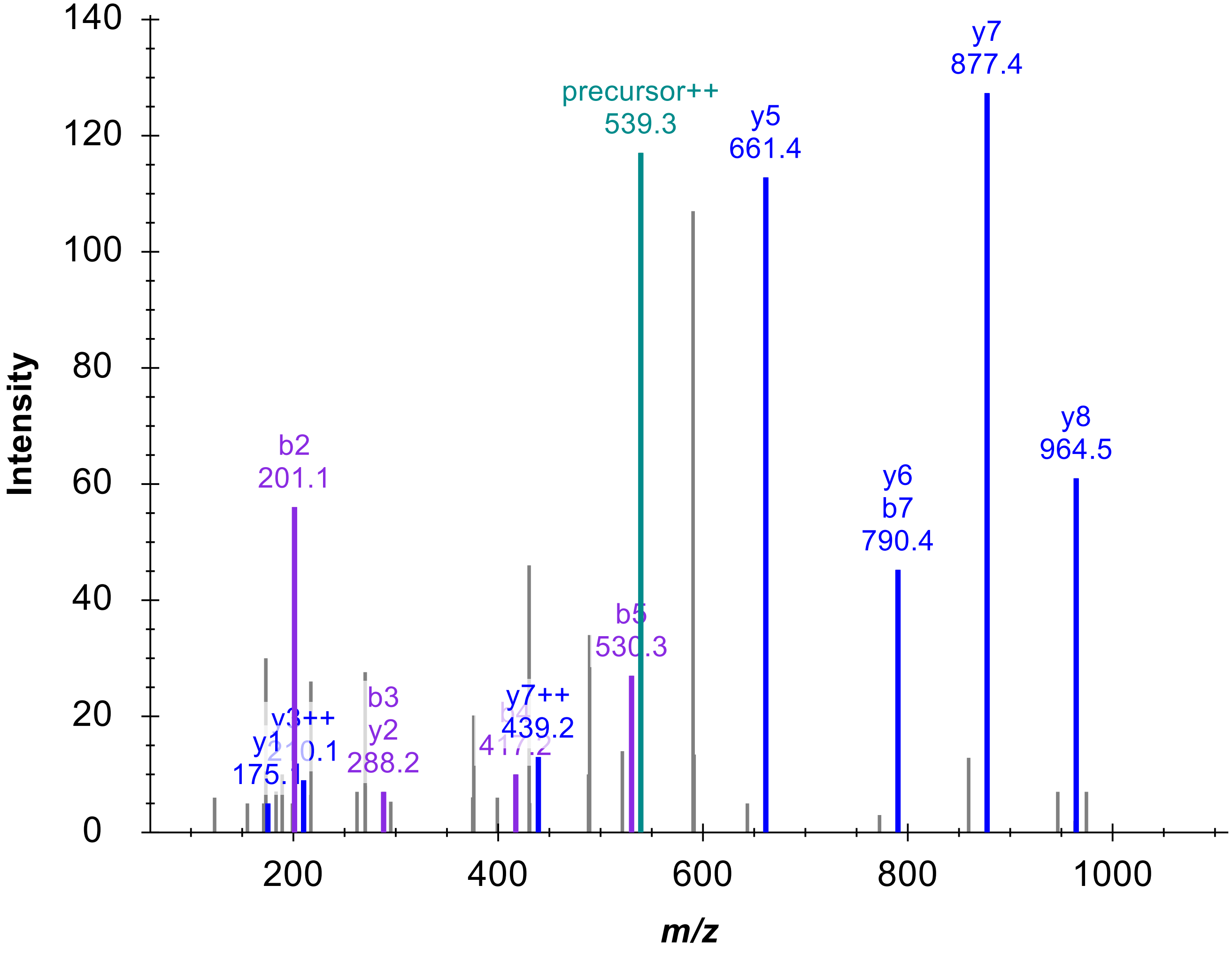

Suppl
Fig 2I

BRD4 Bromodomain-containing protein 4

Q9ESU6 A<u>Q</u>AEHAEKEK [1278, 1287] m/z 571.28 $^{2+}$

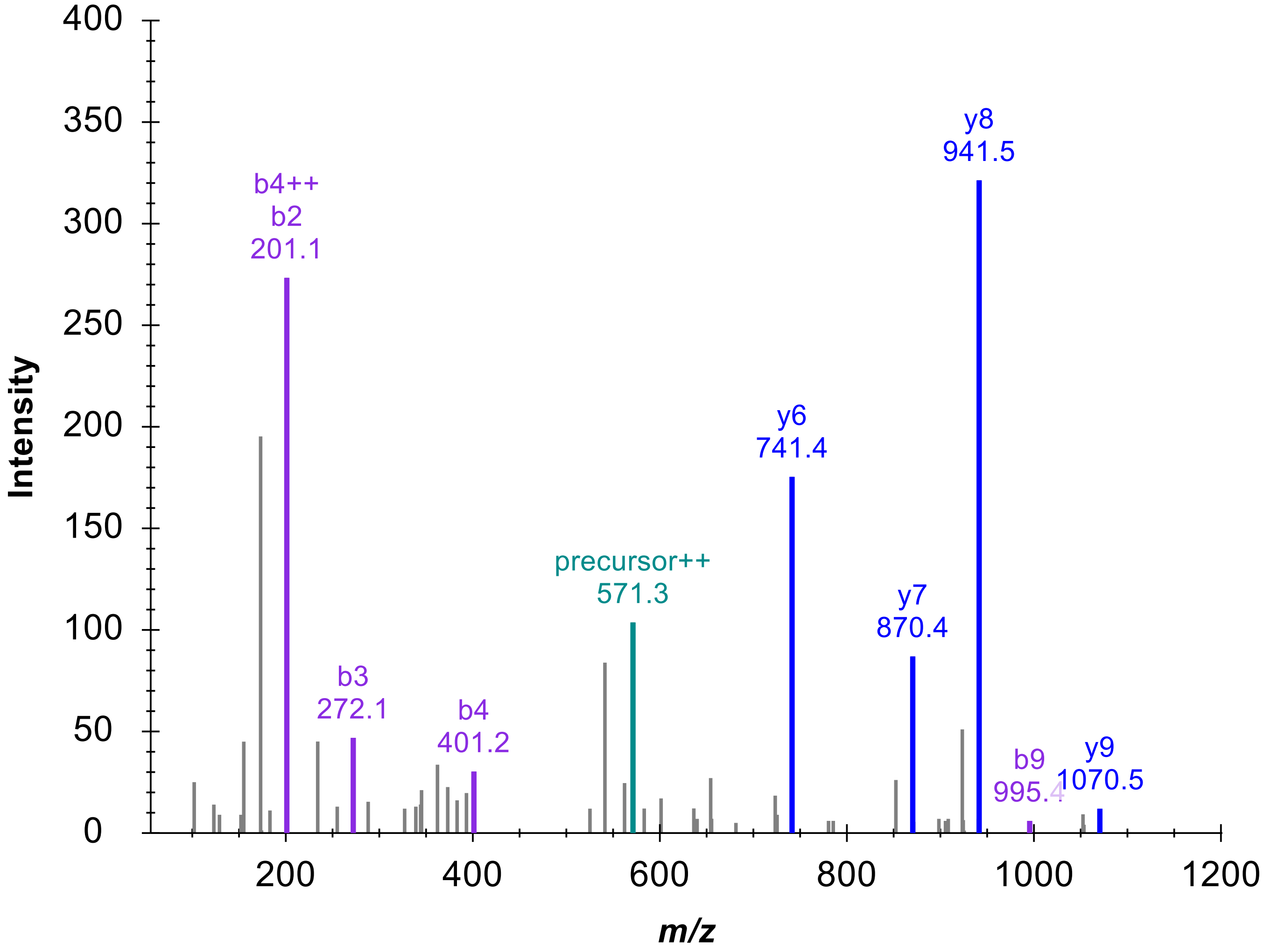

Suppl Fig 2J

PTRF Polymerase I and transcript release factor
Cavin-1

O54724 ATEEPSGTGSDELIK [32, 46] m/z 767.37 $^{2+}$

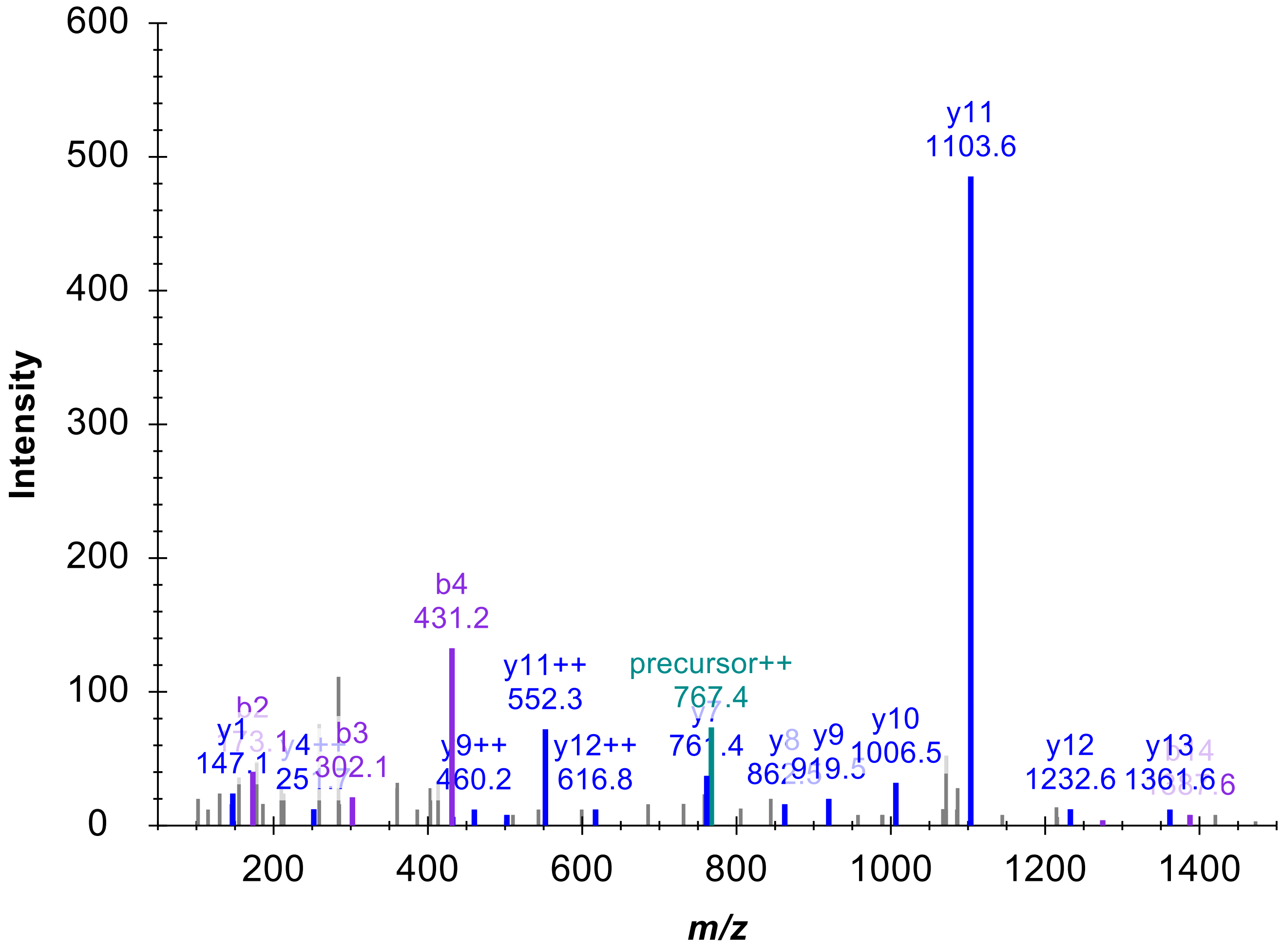

Suppl Fig 2K

SDPR Serum deprivation-response protein
Cavin-2

Q63918 YQASTSNTVSK [102, 112] m/z 593.29 $^{2+}$

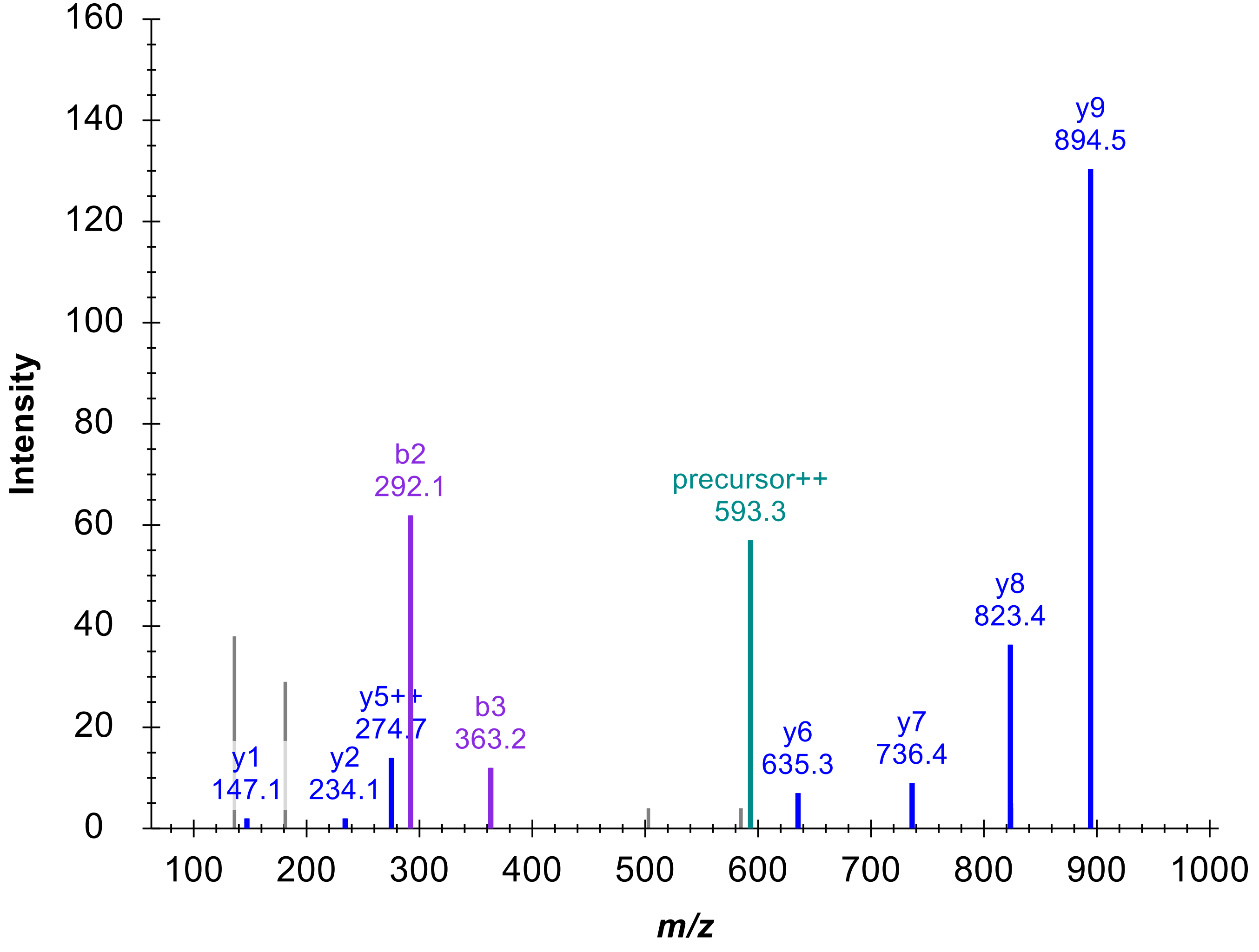

Suppl Fig 2L

MURC Muscle-related coiled-coil protein
Cavin-4

A2AMM0 VASVVDSVQASQKR [39, 52] m/z 491.94 3+

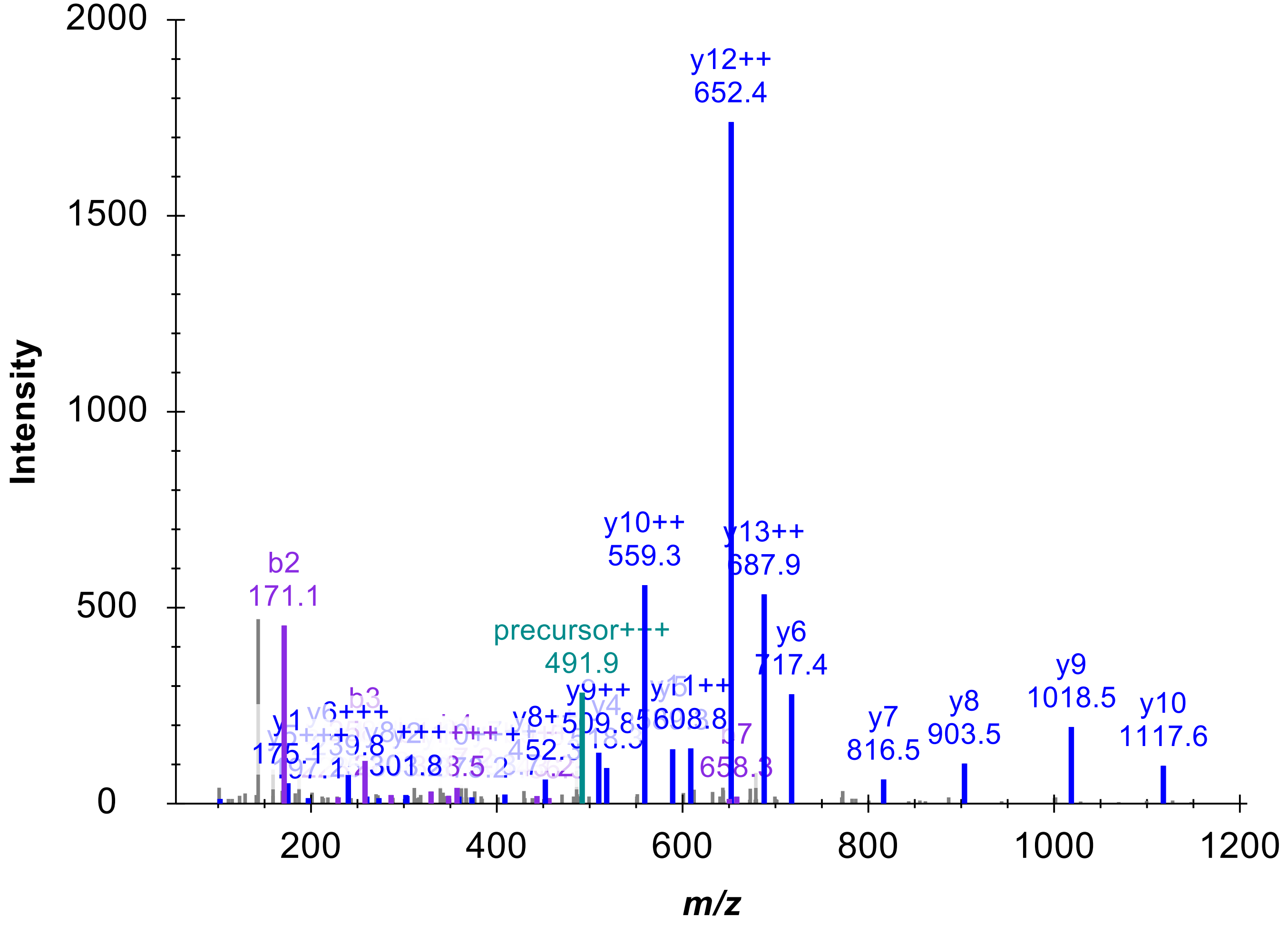

Suppl
Fig 2M

SYNCI Syncoilin

Q9EPM5 ALQDTQAEIQE*M*R [348, 360] 774.87 $^{2+}$

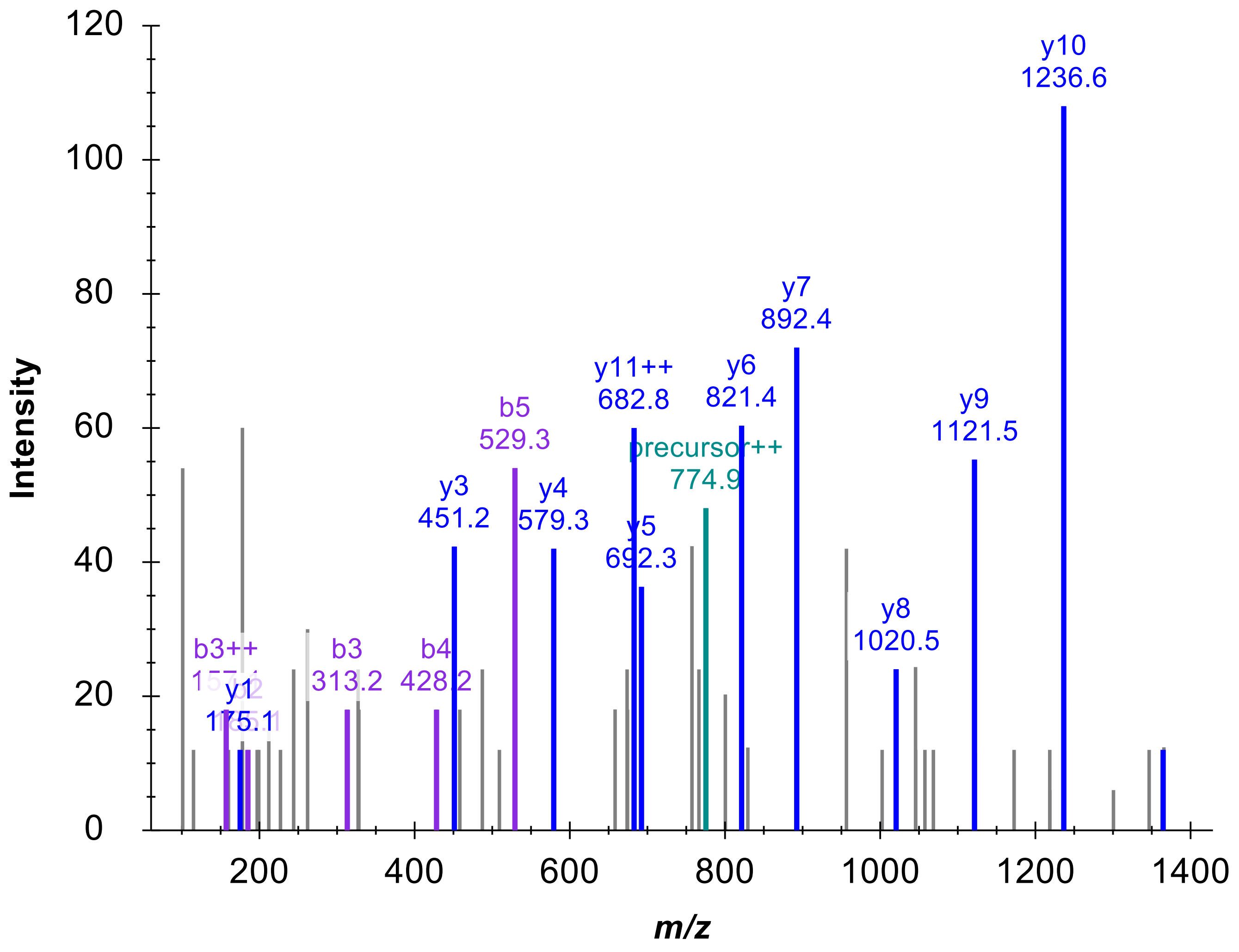

Figure S3

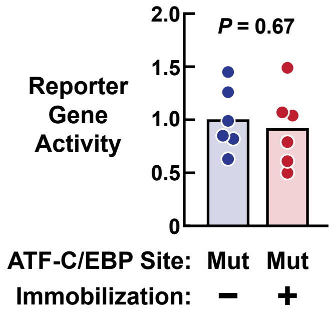
P = 0.67
2.0
1.5
1.0
0.5
0
Reporter Gene Activity
ATF-C/EBP Site: Mut Mut
Immobilization: − +
